# A randomized, double-blind, crossover study of acute low-level night-time road traffic noise: effects on vascular function, sleep, and proteomic signatures in healthy adults

**DOI:** 10.1093/cvr/cvag028

**Published:** 2026-02-25

**Authors:** Omar Hahad, Patrick Foos, Jonas Hübner, Christina Große-Dresselhaus, Frank P Schmidt, Mir Abolfazl Ostad, Marin Kuntic, Lukas Hobohm, Karsten Keller, Volker H Schmitt, Thomas Köck, Philipp Wild, Irene Schmidtmann, Mette Sørensen, Martin Röösli, Paul Stamm, Alexander von Kriegsheim, Johannes Herzog, Philipp Lurz, Andreas Daiber, Thomas Münzel

**Affiliations:** Department of Cardiology, University Medical Center of the Johannes Gutenberg-University Mainz, Langenbeckstraße 1, Mainz 55131, Germany; German Center for Cardiovascular Research (DZHK), Partner Site Rhine-Main, Langenbeckstraße 1, Mainz 55131, Germany; Department of Cardiology, University Medical Center of the Johannes Gutenberg-University Mainz, Langenbeckstraße 1, Mainz 55131, Germany; Department of Cardiology, University Medical Center of the Johannes Gutenberg-University Mainz, Langenbeckstraße 1, Mainz 55131, Germany; Department of Cardiology, University Medical Center of the Johannes Gutenberg-University Mainz, Langenbeckstraße 1, Mainz 55131, Germany; Department of Cardiology, University Medical Center of the Johannes Gutenberg-University Mainz, Langenbeckstraße 1, Mainz 55131, Germany; Department of Internal Medicine 3: Cardiology, Diabetology, Angiology, Klinikum Mutterhaus der Borromäerinnen, Trier, Germany; Department of Cardiology, University Medical Center of the Johannes Gutenberg-University Mainz, Langenbeckstraße 1, Mainz 55131, Germany; Department of Internal Medicine, Kreisklinik Groß-Gerau, Groß-Gerau, Germany; Department of Cardiology, University Medical Center of the Johannes Gutenberg-University Mainz, Langenbeckstraße 1, Mainz 55131, Germany; German Center for Cardiovascular Research (DZHK), Partner Site Rhine-Main, Langenbeckstraße 1, Mainz 55131, Germany; Department of Cardiology, University Medical Center of the Johannes Gutenberg-University Mainz, Langenbeckstraße 1, Mainz 55131, Germany; Center for Thrombosis and Hemostasis, University Medical Center of the Johannes Gutenberg-University Mainz, Mainz, Germany; Department of Cardiology, University Medical Center of the Johannes Gutenberg-University Mainz, Langenbeckstraße 1, Mainz 55131, Germany; Center for Thrombosis and Hemostasis, University Medical Center of the Johannes Gutenberg-University Mainz, Mainz, Germany; Department of Cardiology, University Medical Center of the Johannes Gutenberg-University Mainz, Langenbeckstraße 1, Mainz 55131, Germany; Department of Cardiology, University Medical Center of the Johannes Gutenberg-University Mainz, Langenbeckstraße 1, Mainz 55131, Germany; Preventive Cardiology and Preventive Medicine, Department of Cardiology, University Medical Center of the Johannes Gutenberg-University Mainz, Mainz, Germany; Department of Cardiology, University Medical Center of the Johannes Gutenberg-University Mainz, Langenbeckstraße 1, Mainz 55131, Germany; German Center for Cardiovascular Research (DZHK), Partner Site Rhine-Main, Langenbeckstraße 1, Mainz 55131, Germany; Center for Thrombosis and Hemostasis, University Medical Center of the Johannes Gutenberg-University Mainz, Mainz, Germany; Preventive Cardiology and Preventive Medicine, Department of Cardiology, University Medical Center of the Johannes Gutenberg-University Mainz, Mainz, Germany; Institute of Medical Biostatistics, Epidemiology and Informatics, University Medical Center of the Johannes Gutenberg-University Mainz, Mainz, Germany; Work, Environment and Cancer, Danish Cancer Institute, Copenhagen, Denmark; Department of Natural Science and Environment, Roskilde University, Roskilde, Denmark; Swiss Tropical and Public Health Institute, Allschwil, Switzerland; University of Basel, Basel, Switzerland; Department of Cardiology, University Medical Center of the Johannes Gutenberg-University Mainz, Langenbeckstraße 1, Mainz 55131, Germany; German Center for Cardiovascular Research (DZHK), Partner Site Rhine-Main, Langenbeckstraße 1, Mainz 55131, Germany; Cancer Research UK (CRUK) Scotland Centre, Institute of Genetics and Cancer, University of Edinburgh, Edinburgh, UK; Department of Cardiology, University Medical Center of the Johannes Gutenberg-University Mainz, Langenbeckstraße 1, Mainz 55131, Germany; Department of Cardiology, University Medical Center of the Johannes Gutenberg-University Mainz, Langenbeckstraße 1, Mainz 55131, Germany; German Center for Cardiovascular Research (DZHK), Partner Site Rhine-Main, Langenbeckstraße 1, Mainz 55131, Germany; Department of Cardiology, University Medical Center of the Johannes Gutenberg-University Mainz, Langenbeckstraße 1, Mainz 55131, Germany; German Center for Cardiovascular Research (DZHK), Partner Site Rhine-Main, Langenbeckstraße 1, Mainz 55131, Germany; Department of Cardiology, University Medical Center of the Johannes Gutenberg-University Mainz, Langenbeckstraße 1, Mainz 55131, Germany; German Center for Cardiovascular Research (DZHK), Partner Site Rhine-Main, Langenbeckstraße 1, Mainz 55131, Germany

**Keywords:** Road traffic noise, Endothelial function, Sleep disturbance, Cardiovascular stress response, Proteomics

## Abstract

**Aims:**

Road traffic noise is the dominant source of environmental noise in Europe and a recognized cardiovascular risk factor, yet direct mechanistic evidence from human studies remains limited. This study investigated the acute effects of low-level night-time road traffic noise exposure on cardiovascular parameters in healthy adults.

**Methods and results:**

In a randomized, double-blind, crossover design, 74 healthy participants were exposed to three overnight conditions: control (no noise, average sound pressure level (LAeq) 30.70 dB), 30 (LAeq 41.36), and 60 (LAeq 44.13) recorded road traffic noise events (peak level ≈60 dB). The primary endpoint was endothelial function assessed by flow-mediated dilation (FMD) the morning after each night; a subgroup received vitamin C to assess oxidative stress involvement. Secondary endpoints included sleep quality (questionnaires), cardiovascular parameters (blood pressure, heart rate, electrocardiogram), and targeted proteomic analysis (Olink panels). FMD significantly decreased from 9.35% (control) to 8.19% after 30 noise events (Δ = 1.16%, *P* = 0.005) and 7.73% after 60 events (Δ = 1.63%, *P* < 0.0001), with the strongest FMD improvement by vitamin C in the 60-event condition (Δ = 1.02%). Noise exposure increased heart rate (mean difference Δ = 1.23 bpm, *P* = 0.04; max Δ = 7.95 bpm, *P* < 0.001) and the odds of post-noise heart rate peaks (odds ratio 2.42, 95% confidence interval 2.07–2.83). After noise exposure, self-reported sleep quality and restfulness were significantly impaired across all dimensions. Clinical chemistry blood parameters did not change significantly. Proteomic analysis revealed noise-associated changes in interleukin signalling and chemotaxis in participants with the strongest FMD impairments.

**Conclusion:**

Acute exposure to night-time road traffic noise leads to measurable changes in cardiovascular health parameters in healthy adults. These effects were linked to activation of molecular pathways of immune signalling. Plasma proteome changes were correlated to FMD changes (responders vs. non-responders), highlighting interindividual biological susceptibility to noise.

## Graphical Abstract

**Graphical Abstract:**
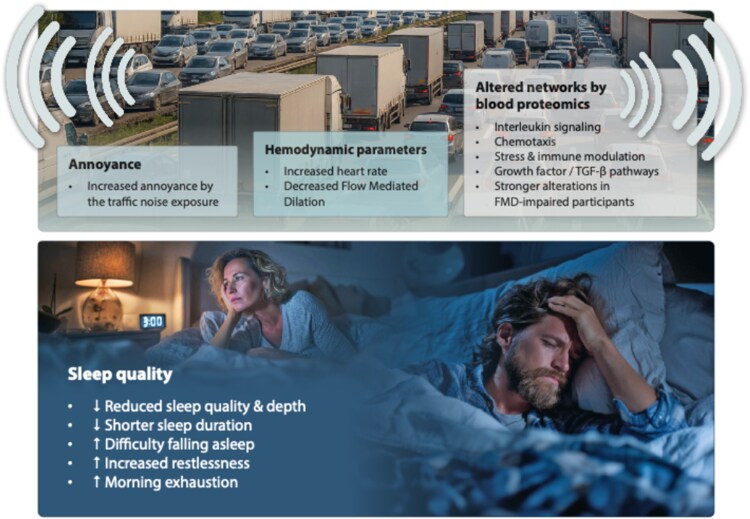

**Time of primary review: 49 days**

## 1. Introduction

Noise pollution is a widespread environmental stressor and a significant public health issue, with road traffic being the primary noise source in the European Union (EU). According to recent European Environment Agency (EEA) estimates, approximately 150 million people in the EU are exposed to harmful levels of road traffic noise, with at least 30% of the population exposed above the guideline values for transportation from the World Health Organization. Long-term exposure is linked to a substantial burden of disease in Europe, including 82 400 deaths, 62 600 cases of cardiovascular disease, 28 100 cases of diabetes, 21 million individuals experiencing high annoyance, and 7 million suffering from chronic sleep disturbance, adding up to 1.7 million disability-adjusted life years (DALYs) attributable to transportation noise each year. Additionally, it is estimated to contribute to 80 300 children having behavioural problems, 608 100 children with reading impairment, and 344 000 children with overweight, as well as 299 600 adults suffering from depressive disorders and 18 700 from dementia due to transportation noise.^1^ Reducing noise pollution is a key objective of the EU’s Zero Pollution Action Plan and the Environmental Noise Directive. The Zero Pollution vision for 2050 includes a 2030 target to reduce the proportion of people chronically disturbed by transportation noise by 30%.^2^ However, a 2023 EU Commission report on the END highlights that, despite two decades of implementation, environmental noise remains a major public health concern, contributing to cardiovascular disease, sleep disturbance, and annoyance.^3^

In recent years, high-quality epidemiological studies based on large, well-characterized cohorts have provided strong evidence for an association between transportation noise exposure and adverse cardiovascular outcomes (for an overview, see.^4,5^) For instance, in a nationwide Danish cohort, a 10-year average exposure to road traffic noise (L_den_), per 10 dB increase, was associated with a hazard ratio of 1.09 [95% confidence interval (CI) 1.08–1.10] for cardiovascular mortality.^6^ In the same cohort, road traffic noise was associated with higher risk of ischemic heart disease, myocardial infarction, angina pectoris, and heart failure, with hazard ratios of 1.052 (1.044–1.059), 1.041 (1.032–1.051), 1.095 (1.071–1.119), and 1.039 (1.033–1.045), respectively, per 10 dB higher 10-year mean exposure.^7^ While it is widely established that transportation noise may contribute to cardiovascular risk by triggering noise annoyance, interfering with daily activities and sleep, and increasing mental distress, ultimately leading to physiological arousal through the so-called non-auditory pathway,^8^ direct human experimental evidence remains limited. Such studies are essential to disentangle the pathophysiological mechanisms underlying noise-induced cardiovascular disease. Most human mechanistic insights stem from experimental studies investigating the acute effects of night-time aircraft^9–11^ or train noise^12^ on cardiovascular function, consistently demonstrating that noise exposure induces endothelial dysfunction, blood pressure dysregulation, stress hormone release, and impaired sleep. Notably, these effects appear to be even more pronounced in individuals with prevalent or at increased risk for coronary artery disease.^10^ These findings align with extensive translational research in animal models, which has identified inflammation and oxidative stress as key drivers of noise-induced cardiovascular pathology.^13,14^

To date, there is a lack of controlled human experimental evidence providing detailed mechanistic insights into the cardiovascular sequelae of acute road traffic noise exposure, which is important because road traffic noise is the predominant source of environmental noise affecting large segments of the population. We conducted a randomized, double-blind, crossover study to investigate the acute effects of night-time road traffic noise exposure on endothelial function, cardiovascular health, sleep, and proteomics in healthy individuals. Additionally, we evaluated the potential role of oxidative stress in mediating the effects of noise on endothelial function by administering the antioxidant vitamin C to a subgroup of participants. The average sound pressure levels (LAeq) used in the present study were below those applied in our previous human field studies on aircraft noise (LAeq up to 46.3 dB)^9^ and railway noise (LAeq up to 54.5 dB).^12^ Although the average sound pressure level used in the present study is measured indoor and reflects acute exposure, it is in the range that is considered safe for long-term night-time road traffic noise exposure by the World Health Organization (WHO) (<45 dB, measured outdoor at the most exposed facade),^1^ and could be of relevance for people sleeping with open windows in their bedroom. Similar sound pressure levels have been demonstrated to cause significant changes in cardiac structure and function in response to night-time aircraft noise (*L*_night_).^15^

## 2. Methods

### 2.1 Study design and sample

This study aimed to investigate the acute effects of night-time road traffic noise exposure in healthy individuals by subjecting them to three distinct study nights with varying numbers of recorded road traffic noise. The study protocol included a control night without recorded noise and two experimental nights featuring either 30 or 60 road traffic noise events, each reaching peak sound levels of approximately 60 dB. The study followed a randomized, double-blind, crossover design with the order of study nights randomly assigned via computer-generated randomization. Six different randomization sequences of study nights were used: Control–Noise30–Noise60, Control–Noise60–Noise30, Noise30–Control–Noise60, Noise30–Noise60–Control, Noise60–Control–Noise30, and Noise60–Noise30–Control. Neither participants nor investigators involved in data collection were aware of the assigned noise condition at the start of each study night.

The primary endpoint was assessment of endothelial function, measured by flow-mediated dilation (FMD) of the brachial artery. FMD was chosen as it serves as an early biomarker of cardiovascular risk and endothelial dysfunction.^16^ To investigate the potential involvement of oxidative stress in endothelial responses to recorded road traffic noise, a secondary endpoint examined the impact of high-dose vitamin C (2 g) on FMD. Following the initial FMD measurement on the morning after each study night, 31 participants received an oral dose, whereas 43 participants had no intervention, after which a second FMD measurement was taken 2 h later. The relative change in FMD was then calculated to assess the potential antioxidative effects of vitamin C. Furthermore, the study recorded a range of physiological parameters indicative of cardiovascular health and questionnaire information collected using a standardized evening-to-morning protocol (see description later).

### 2.2 Recruitment, screening, and inclusion

Participant recruitment, screening, and inclusion followed strict methodological and ethical standards. The study was conducted in accordance with the Declaration of Helsinki, with ethical approval granted by the Ethics Committee of the State Medical Association of Rhineland-Palatinate (application number 2020-15345). Written informed consent was obtained from all participants before inclusion. Based on previous human experimental studies and power/sample size calculations, a minimum of 70 individuals was determined to be necessary to include.^9–12^ Participant recruitment began in April 2021, with subsequent measurements conducted between July 2021 and May 2023. Recruitment was conducted primarily through social media, flyers, and newspaper advertisements. After an initial screening via phone or email, eligible individuals attended an in-person screening at the study centre (Department of Cardiology, University Medical Center, Mainz, Germany). Only healthy, non-smoking individuals aged 18–60 years were invited. Individuals underwent a comprehensive medical examination, including measurements of anthropometrics, blood chemistry, blood pressure, and heart rate. Standardized questionnaires were used to assess exclusion criteria, which included pre-existing medical conditions, indications of sleep disorders or obstructive sleep apnoea, regular medication use, current shift work, residence in high-noise environments, pregnancy, and age-adjusted hearing loss higher than 30 dB.

### 2.3 Study nights

**Figure 1:**
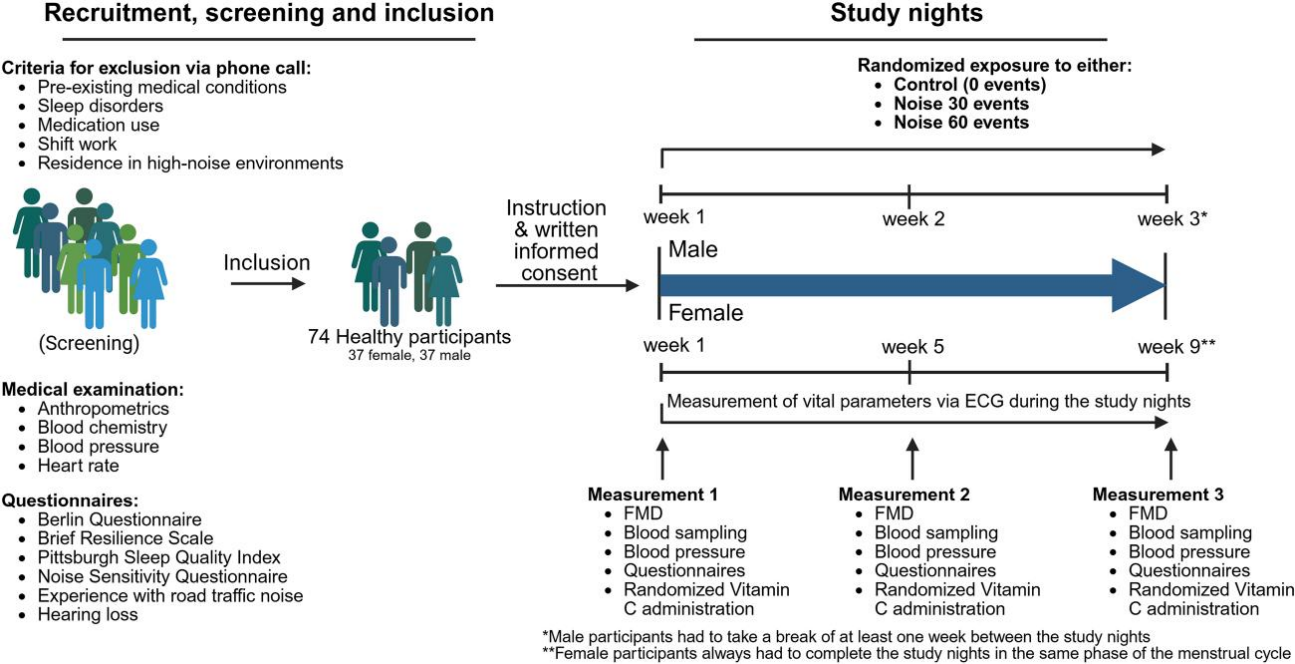
Study design and procedures.

To minimize potential confounding, participants were required to abstain from alcohol, caffeine, nicotine, recreational substances, and strenuous physical activity throughout the study period while adhering to their habitual routines. On each study day, they received calibrated monitoring equipment (SOMNOtouch NIBP[non-invasive blood pressure]; SOMNOmedics GmbH, Randersacker, Germany) and were instructed to ensure a minimum of 7 h in bed. Prior to sleep, they completed an evening protocol, positioned the sound level meter and playback device according to standardized specifications, verified electrode placement, and recorded evening blood pressure. Participant compliance with night-time noise playback was verified through continuous sound level recording. Each participant used a calibrated sound level meter placed at head height that continuously recorded environmental noise throughout the night. Study staff analysed these files the following morning, and characteristic peak patterns were used to confirm full playback and correct exposure duration. Deviations greater than five minutes or atypical sound profiles would have led to exclusion. In addition, participants recorded bedtime, wake-up time, and device status in nightly reports, which were cross-checked for consistency. No non-compliance occurred. All sound files commenced with a standardized 30-s calibration tone followed by a 40-min silent period. Night-time road traffic noise was simulated using two pre-recorded sound files (≈6.5 h each), differing only in event frequency (30 vs. 60 events). These sound files used in the present study were real-life recordings and were recorded by a professional sound engineer. Each noise event lasted 1 min 15 s, with inter-event intervals of 575 s (60-event condition) or 700 s (30-event condition). Events comprised continuous road traffic noise with equal peak levels, and noise intensity was calibrated based on A-weighted equivalent continuous sound levels (LAeq). The sound files were generated using Audacity (https://www.audacityteam.org). Male participants had a 1-week washout period between study nights, while female participants had a 4-week interval to account for hormonal variations. The following morning, participants recorded blood pressure, completed a morning protocol, and attended follow-up assessments at the study centre in a fasting state. Participants lived in or near Mainz, allowing short travel times to the study centre. All returned the next morning between 07:00 and 09:00 a.m., approximately 60 to 120 min after awakening. A minimum in-bed time of 7 h was required and verified by continuous sound and vital recordings. Each participant had a fixed morning appointment with the same staff member, and punctuality was closely monitored to ensure standardized conditions and to minimize circadian or activity-related confounding. Follow-up assessments included measurements of FMD, blood pressure, blood sampling, and verification of questionnaire data. Participants received a total compensation of €120 (€10 for screening, €30 per study night, and €20 upon study completion). *Figure 1* gives an overview of the study design and procedures.

### 2.4 Measurements during study period

#### 2.4.1 Flow-mediated dilation

FMD measurements were conducted by trained study nurses and technicians at the study centre in accordance with previous studies.^9–12^ Each participant was assigned to a specific examiner to minimize variability between conditions. The examination environment was standardized, with dimmed lighting and a comfortable examination set-up. A resting period of 5 min preceded each measurement. FMD was measured using a Philips Affiniti 70G ultrasound device with a 5–12 MHz transducer and an SC5 cuff (Hokanson, Bellevue, WA). Data were processed with Cardiovascular Suite Ultrasound Edition software (version 2.7.0, Quipu, Pisa, Italy).

#### 2.4.2 Road traffic noise playback

Road traffic noise during study nights was played back using a SONY ZS-PS50 system. The playback and recording set-up was standardized using a detailed instruction manual provided to each participant. The playback device was placed at the foot of the bed, approximately 1 m above the floor, while the sound level meter (EXTECH model 407780A) was positioned near the head of the bed at comparable height. A minimum distance of ∼2 m between devices was ensured in all settings. The instructions were reviewed in person with each participant, and doctoral staff were available 24 h for technical questions. Compliance was again verified by the recorded sound profiles. Randomized sound files (A, B, C) corresponded to experimental nights with 30 and 60 noise events, as well as the control night without recorded road traffic noise.

#### 2.4.3 Monitoring of physiological vital parameters

The SOMNOtouch NIBP device (SOMNOmedics GmbH, Randersacker, Germany) was used to monitor vital parameters during study nights. Calibration included participant-specific data, baseline blood pressure, electrocardiography, and pulse oximetry. The device automatically recorded data during the study nights. A rechargeable lithium-ion battery powered the device, and data were analysed with Domino light software (version 1.5.0.11).

#### 2.4.4 Blood pressure monitoring

Evening and morning blood pressure measurements were self-administered using the pre-calibrated boso-medicus uno device (Bosch+Sohn GmbH u. Co. KG, Jungingen, Germany). Study staff provided standardized instructions to ensure reliable use.

#### 2.4.5 Blood chemistry analysis

Peripheral venous blood samples were collected at screening and after each study night in a fasting state for routine blood chemistry analysis. Blood samples were processed using a Hettich EBA 200 centrifuge and stored at −80°C. Analyses followed the standards of the University Medical Center’s central laboratory.

#### 2.4.6 Questionnaires and further assessments

Participants completed a range of validated questionnaires and assessments during the screening to determine their eligibility for inclusion in the study in line with previous studies.^9–12^ These included evaluations of residential night-time noise exposure (with exclusion for levels exceeding 45 dB), as well as assessments of attitudes and experiences related to road traffic noise, sleep patterns, and health status. Specific exclusion criteria during screening were chronic cardiovascular conditions (e.g. coronary heart disease, prior thromboembolic events, diabetes mellitus, arterial hypertension), chronic kidney or nervous system diseases (e.g. epilepsy), current pregnancy, regular medication or substance use (e.g. alcohol, tobacco), regular night shift work, and sleep apnoea. Additionally, participants were required to pass a hearing test during screening; hearing loss greater than 30 dB(A) in either ear led to exclusion. Age-adjusted hearing loss was also assessed to ensure eligibility. For participants included in the study, additional assessments were conducted during the study nights to document subjective road traffic noise annoyance, sleep quality, and well-being on the basis of an evening-to-morning protocol. These procedures were accompanied by the use of the Berlin Questionnaire,^17^ Pittsburgh Sleep Quality Index,^18^ Noise Sensitivity Questionnaire,^19^ experience with road traffic noise questionnaire, Brief Resilience Scale,^20^ and Morningness–Eveningness Questionnaire^21^ were used during the self-reported data were reviewed for completeness by study personnel.

### 2.5 Targeted proteomics

To address the role of aircraft noise exposure on the severity of coronary artery disease and thromboinflammation, the 179 CVD- and inflammation-related human protein biomarkers of the Olink Target 96 CVDII and INFLAMMATION panels were measured based on the Proximity Extension Assay (PEA) technology (Olink Biosciences, Uppsala, Sweden), as described elsewhere.^12,22,23^ In brief, once-thawed ethylenediaminetetraacetic acid (EDTA)-blood plasma was used for analysis. For each target antigen, the affinity-based PEA technique uses a pair of antibodies linked to unique, partially complementary single-stranded DNA oligonucleotides. After the simultaneous binding of both antibodies to an antigen molecule, close proximity allows for the formation of a PCR target sequence by hybridization. After unspecific preamplification, amplicons were quantified by qPCR using protein-specific primer pairs. The resulting *C_t_*-value of each protein (Fluidigm Real-Time PCR Analysis Software, Version 4.3.1, San Francisco, CA) was transformed to normalized protein expression (NPX) units using software from the manufacturer (Olink NPX Manager, Version 1.1.4.0, Uppsala, Sweden). NPX units represent relative quantifications of protein concentrations on a log2-scale (i.e. an increase by one NPX represents a duplication of protein concentration).

### 2.6 Bioinformatic analysis of proteomic data

For analysis, subject samples were grouped, and differential expression analysis was performed using the Perseus software suite. Briefly, expression values were log2 transformed, PCA plots were generated, and differential expression was determined by applying the Student's *t*-test using a cut-off of *P* < 0.05. Proteins that were differentially expressed in at least one group were retained. To visualize expression changes across the groups, we determined the mean value, *Z*-normalized, and proteins were clustered using Euclidean distance and visualized as heat maps. Network analysis and data representation were performed using Cytoscape. Differentially expressed proteins were mapped onto a protein–protein interaction network generated by String. Edges represent interactions based on genetic, experimental, biochemical, and text-mining data using a threshold of >0.4 for the interaction score. Of note, the network was not restricted to protein–protein interactions (experiments), the network represents genetic interactions as well as co-mentioned in literature (biological interaction in its broadest sense). For analysis of subgroups, only selected subjects were compared (20 displaying the lowest FMD changes by noise and 20 displaying the highest FMD changes by noise).

### 2.7 Effect of noise peaks on heart rate

**Figure 2:**
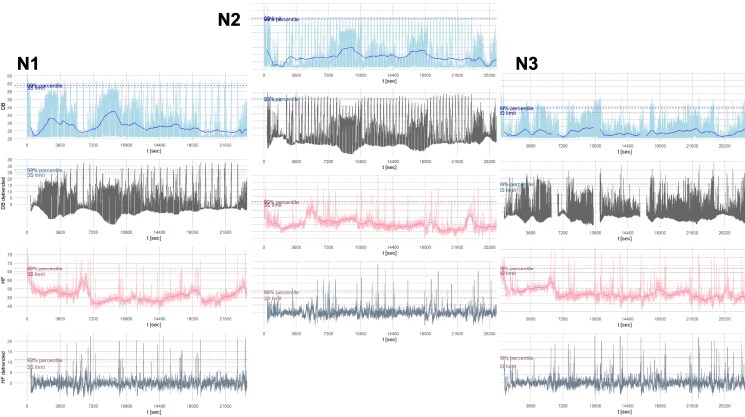
Detection of heart rate responses to noise peaks across study nights (N1 = night 1, N2 = night 2, N3 = night 3). Noise and heart rate were recorded every second during each study night and detrended using a ±350-s moving average. Heart rate responses were assessed by identifying peaks within 180 s after noise peaks and comparing average heart rate before and after each noise event.

During the study nights, noise levels and heart rate were continuously recorded at 1-s intervals. Data from each device were exported in a structured and consistent file format. The datasets were subsequently merged using synchronized timestamps via the Python pandas library (https://pandas.pydata.org/) within Python (https://www.python.org/). For visual assessment, the merged data were plotted using the matplotlib library (https://matplotlib.org/). These data were analysed with the aim to describe immediate effects of noise peaks on heart rate (*Figure 2*). Data for each participant on each night was processed as follows: underlying slow trends for both heart rate and noise level were determined by applying moving averages with a window of ±350 s at each data point. Given the planned intervals between noise events, this yielded a smooth trend and allowed to separate noise peaks—both intended and unintended—from background noise. This is also within the recommended window of 10–15 min for the analysis of heart rate data. A noise peak was postulated when the noise level was above the 99% percentile for a specific participant on a specific night, and there were at least three subsequent values above this threshold. For the heart rate, peaks were obtained from de-trended values. That is, the average heart rate in the interval [*t* − 350, *t* + 350] was subtracted from the observed value at time (*t*) to obtain the de-trended value. A peak in heart rate was postulated when the de-trended value was above the 99% percentile of de-trended values for a specific participant in a specific night, and there were at least three subsequent values above this threshold. For both noise and heart rate, several high values were only considered as separate peaks when they were at least 75 s apart, that is, the duration of the intended noise events. The effect of noise peaks at times *t*_peak_ on heart rate was described in two ways. First, by determining whether a peak of heart rate occurred in an interval [*t*_peak,_  *t*_peak_ + 180 s]. If there were unintended noise peaks, this period was curtailed such that it ended 30 s before the next noise peak. Second, the average heart rate in the interval [*t*_peak_ – 120 s, *t*_peak_ – 60 s] *before* the noise peak was compared to the average heart rate [*t*_peak,_  *t*_peak_ + 60 s] *after* the noise peak.

### 2.8 Statistical analysis

Categorical variables were described by absolute and relative frequencies. Quantitative variables were described by mean, standard deviation, median, minimum, maximum, and quartiles, stratified by exposure. To assess effects of noise exposure on quantitative variables, we fitted linear mixed models with exposure and period, that is, first, second, or third night, as fixed effects and participant as a random effect, thus considering that measures on the same participant are not independent. The difference between estimated means from the model is displayed with 95% confidence interval [CI]. *P*-values for testing the null hypotheses of a null difference between measurements under different exposures are also included. The occurrence of heart rate peaks after noise peaks was modelled using generalized linear mixed models, with binary outcome and logit-link function. The average heart rate before and after noise peaks was analysed using a linear mixed model. In these models, period and exposure were fixed effects, and participant was a random effect. Data were analysed using SAS 9.4 (descriptive statistics and PROC MIXED) and R 4.4.3 (packages zoo, pracma for peak detection, lme4, and emmeans for mixed models). The statistics of the proteome data is described in Section ‘Bioinformatic analysis of proteomic data’.

## 3. Results

A total of 74 participants were included and analysed in this study, after excluding seven individuals who withdrew following enrolment due to insufficient recovery after the study nights, discomfort during FMD measurements, or time constraints related to study completion. Baseline characteristics are presented in *Table 1*. The study sample was balanced in terms of sex and comprised generally young adults with normal anthropometric characteristics. All participants had physiological values for glycaemic control and renal function, as well as lipid parameters within healthy ranges. Questionnaire-based assessments indicated a low likelihood of sleep apnoea, moderate psychological resilience, and overall good sleep quality. Participants reported moderate prior experience with road traffic noise and low to moderate levels of noise sensitivity, including in relation to sleep.

**Table 1:** Baseline characteristics of study participants (*N* = 74)

Variables	Mean ± standard deviation or absolute numbers and relative percentages
Female sex [*n* (%)]	37 (50)
Age (years)	26.1 ± 5.6
Weight (kg)	71.7 ± 13.1
Height (cm)	173.9 ± 9.1
Body mass index (kg/m^2^)	23.6 ± 3.6
Berlin Questionnaire (score, 0–39)	7.7 ± 4.2
Brief Resilience Scale (score, 1–5 points)	3.7 ± 0.7
Experience with road traffic noise questionnaire (score, 0–64 points)	32.2 ± 6.8
Noise Sensitivity Questionnaire (total score, 0–3 points)	1.3 ± 0.3
Noise Sensitivity Questionnaire sleep dimension (score, 0–3 points)	1.18 ± 0.54
Pittsburgh Sleep Quality Index (score, 0–21 points)	3.7 ± 2.0
Glycated haemoglobin (HbA1c) (%)	5.02 ± 0.27
Low-density lipoprotein (mg/dL)	98.6 ± 27.0
High-density lipoprotein (mg/dL)	57.9 ± 13.4
Total cholesterol (mg/dL)	176.5 ± 31.3
Triglycerides (mg/dL)	99.8 ± 50.4
Creatinine (mg/dL)	0.82 ± 0.14

Berlin Questionnaire assessing sleep-wake patterns. Scores >25 indicate high sleep apnoea risk, <15 make it unlikely, and 15–25 suggest a pathological pattern.

Brief Resilience Scale assessing the ability to recover from stress, with scores ranging from 1 to 5 per item. Higher scores indicate greater psychological resilience.

Experience with road traffic noise questionnaire assessing personal experiences and attitudes towards road traffic noise, with higher scores indicating more negative experience.

Noise Sensitivity Questionnaire assessing noise sensitivity across various contexts. Scores range from 0 to 3, with higher scores indicating higher sensitivity.

Pittsburgh Sleep Quality Index assessing subjective sleep habits over the past month. Scores ranging from 0 to 21, with higher scores indicating poorer sleep quality (score >10 led to exclusion).

### 3.1 Primary endpoint: flow-mediated dilation

**Figure 3:**
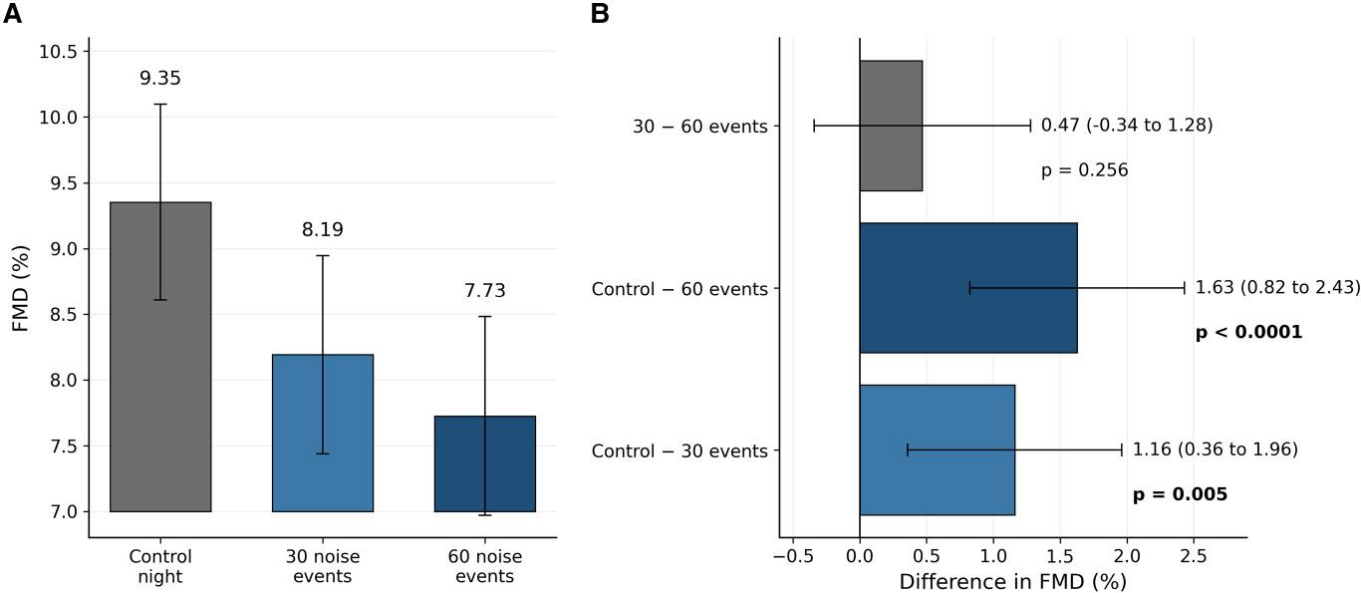
Effects of night-time road traffic noise exposure on the primary endpoint flow-mediated dilation (FMD) (*N* = 219). (*A*) Mean FMD (%) with 95% confidence intervals (CI) following the control night and nights with 30 and 60 recorded road traffic noise events. Values represent model-based estimates from linear mixed models with exposure as fixed effect and participant as a random effect. (*B*) Pairwise differences in FMD (%) between exposure conditions with 95% CI. Displayed are the estimated mean differences between control vs. 30 events, control vs. 60 events, and 30 vs. 60 events (see Supplementary material online, *Table S1* for exact numerical values).

Night-time road traffic noise exposure was associated with a significant reduction in FMD. Mean FMD decreased from 9.35% (95% CI 8.61–10.10) under control conditions to 8.19% (95% CI 7.44–8.95) following 30 road traffic noise events and to 7.73% (95% CI 6.97–8.48) following 60 road traffic noise events (*Figure 3A* and Supplementary material online, *Table S1A*). Compared to control, the reductions were statistically significant for both 30 (Δ = 1.16%, *P* = 0.005) and 60 events (Δ = 1.63%, *P* < 0.0001). There was no significant difference between nights with 30 and 60 road traffic noise events (*P* = 0.256) (*Figure 3B* and Supplementary material online, *Table S1B*).

**Figure 4:**
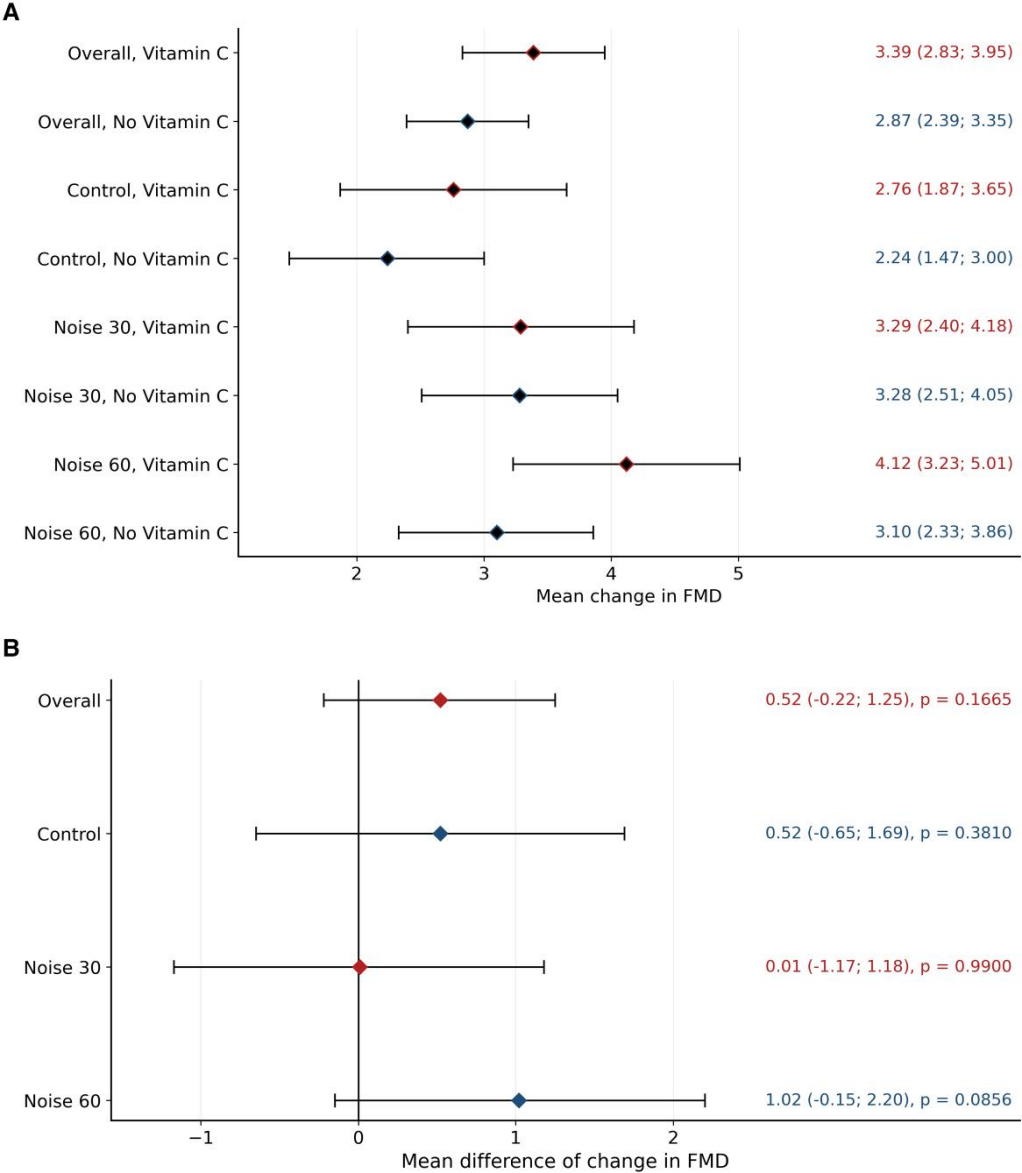
Comparison of changes in flow-mediated dilation (FMD) after vitamin C intake vs. no intake across experimental conditions (*N* = 218). (*A*) Mean changes in flow-mediated dilation (FMD, %) 2 h after vitamin C intake compared with no intake across the three exposure conditions (control, 30 events, 60 events and combined across exposure conditions). (*B*) Group differences in changes in FMD across the three exposure conditions (control, 30 events, 60 events and combined across exposure conditions). Estimates were obtained from linear mixed models including exposure and intervention as fixed effects and participant as a random effect (see Supplementary material online, *Table S2* for exact numerical values).

In the subgroup analysis evaluating vitamin C effects (*Figure 4* and Supplementary material online, *Table S2*), the difference in FMD change between vitamin C and no intervention was statistically not significant across conditions. However, a trend was observed at 60 road traffic noise events (Δ = 1.02%, *P* = 0.09).

### 3.2 Noise annoyance and sleep outcomes

**Figure 5:**
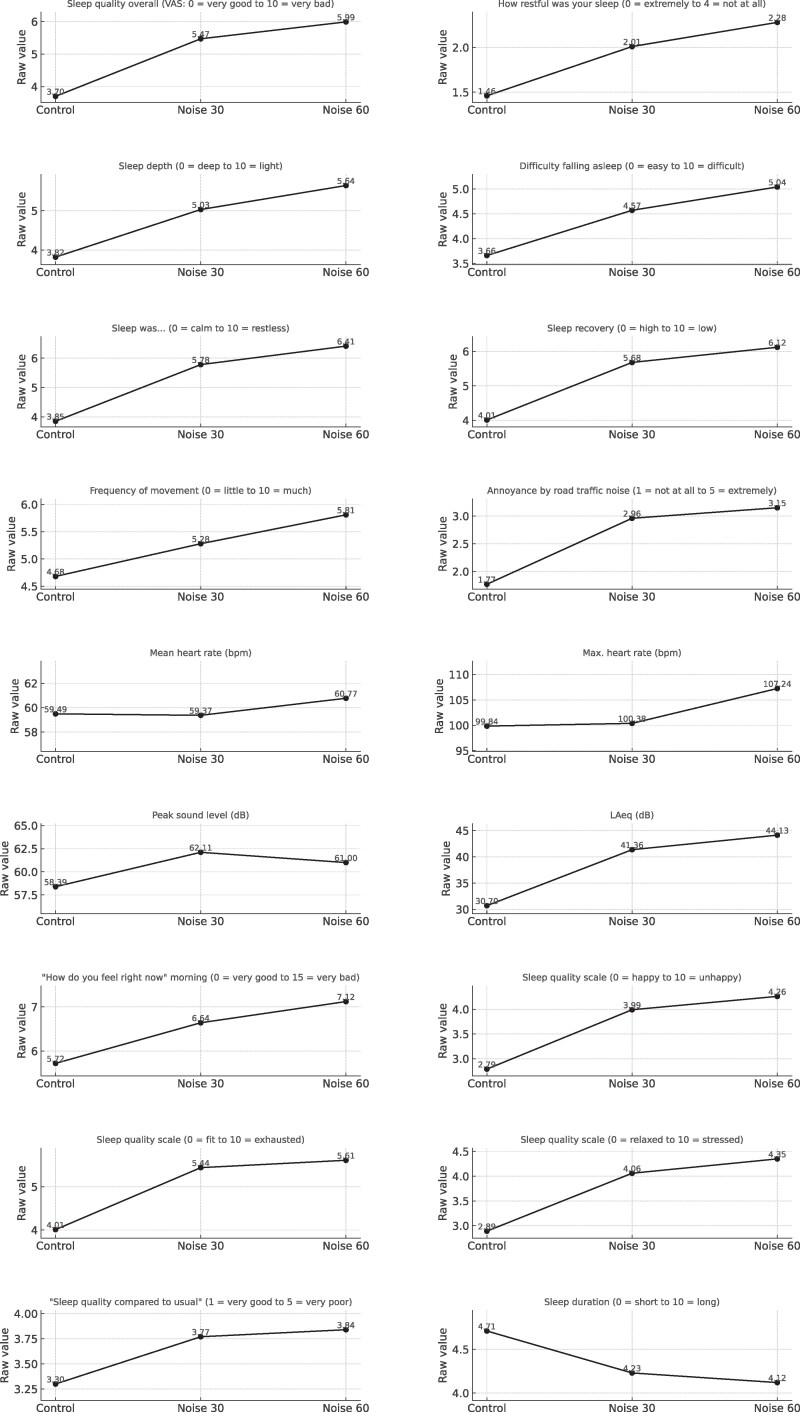
Associations of night-time road traffic noise exposure with secondary endpoints. Shown are only those endpoints with statistically significant differences (*P* < 0.05) between noise exposure conditions, based on a mixed linear model with subjects as a random effect and exposure (control, 30, and 60 noise events) as a fixed effect. The number of observations (n) for each outcome can be obtained from Supplementary material online, *Table S3*, which also presents exact estimates and full results across all endpoints.

Road traffic noise exposure was associated with a significant increase in road traffic noise annoyance and a deterioration in all assessed dimensions of subjective sleep quality (*Figure 5* and Supplementary material online, *Table S3*). Participants rated their sleep as significantly less restful during both noise conditions compared to control. Overall sleep quality, sleep recovery, sleep depth, restlessness, and difficulty falling asleep were all adversely affected, with statistically significant differences between control and both 30 and 60 road traffic noise events. The number of movements during sleep and the perception of sleep as more exhausting and stressful increased with road traffic noise exposure. For sleep quality, participants rated their sleep as worse than normal in both noise conditions.

### 3.3 Blood chemistry

No statistically significant differences were observed in circulating markers of inflammation, stress, or metabolic function across noise exposure conditions (see Supplementary material online, *Table S3*). Levels of adrenaline, interleukin (IL)-6, cortisol, glucose, C-reactive protein (CRP), and neutrophil granulocytes remained stable between the control, 30-, and 60-event study nights. While adrenaline levels showed a non-significant increase and CRP values a slight trend towards reduction across the study nights, none of the changes reached statistical significance.

### 3.4 Hemodynamic parameters

No statistically significant differences in systolic or diastolic blood pressure measured before FMD at the study centre were observed between exposure conditions (see Supplementary material online, *Table S3*). During the study nights, average heart rate was significantly higher in the 60-event condition compared to control (Δ = 1.23 bpm, *P* = 0.04), while no significant difference was observed between control and the 30-event condition. Maximum heart rate was also significantly elevated in the 60-event condition compared to both control (Δ = 7.95 bpm, *P* < 0.001) and the 30-event condition (Δ = 7.38 bpm, *P* < 0.001) (*Figure 5* and Supplementary material online, *Table S3*).

### 3.5 Effect of noise peaks on heart rate

Thirty-seven participants had evaluable data in the control night, 44 participants had evaluable data for exposure with 30 road traffic noise events, and 39 participants had evaluable data for exposure with 60 road traffic noise events.

Peaks in heart rate are more likely to occur after noise peaks in nights without intentional noise events than in nights with intentional noise events, for example, comparing control nights to nights with 30 road traffic noise events resulted in an odds ratio (OR) of 1.89 (95% CI 1.59–2.24) and 2.42 (95% CI 2.07–2.83) when compared to nights with 60 road traffic noise events (*Table 2*). This effect was most pronounced in the first study period (night) and least pronounced in the second period.

**Table 2:** Comparison of heart rate peak occurrence after noise peaks across study nights

			95% confidence interval		
Comparison	Period	Odds ratio	Lower limit	Upper limit	*P-*value
Control/Noise 30	1	2.61	1.96	3.49	**<0.001**
Control/Noise 30	2	1.51	1.08	2.10	**0.015**
Control/Noise 30	3	1.70	1.23	2.34	**0.001**
Control/Noise 30	all	1.89	1.59	2.24	**<0.001**
Control/Noise 60	1	3.28	2.40	4.49	**<0.001**
Control/Noise 60	2	1.90	1.45	2.49	**<0.001**
Control/Noise 60	3	2.27	1.71	3.01	**<0.001**
Control/Noise 60	all	2.42	2.07	2.83	**<0.001**
Noise 30/Noise 60	1	1.25	0.90	1.75	0.183
Noise 30/Noise 60	2	1.26	0.90	1.76	0.182
Noise 30/Noise 60	3	1.34	0.98	1.83	0.070
Noise 30/Noise 60	all	1.28	1.07	1.54	**0.007**

Estimates were derived from a generalized linear mixed model with subjects as random effects and exposure (i.e. control scenario, 30 road traffic noise events, and 60 road traffic noise events) and period as fixed effects. Period refers to the chronological order of the study nights in the crossover design (i.e. period 1 = first night, period 2 = second night, period 3 = third night), independent of the assigned exposure condition. This allows adjustment for potential order effects. Thirty-seven participants had evaluable data for the control night, 44 for the 30-event night, and 39 participants for the 60-event night. Statistically significant *P-*values (i.e. < 0.05) are presented in bold.

Mean heart rate in a 1-min interval after a noise peak is, on average, higher than heart rate in a 1-min interval before the same noise peak. Higher increases in heart rate were observed in control nights than in nights with road traffic noise events. The average increase in control nights was 1.35 bpm (95% CI 1.00–1.70) higher than in nights with 30 traffic noise events and 1.61 bpm (95% CI 1.30–1.91) higher than in nights with 60 road traffic noise events (*Table 3*).

**Table 3:** Average change (increase) in heart rate after noise peaks by exposure and pairwise comparisons between exposure conditions

				95% Confidence interval	
Exposure	Period	Mean	Standard error	Lower limit	Upper limit
Control	1	3.12	0.24	2.65	3.58
Control	2	1.42	0.22	0.98	1.86
Control	3	1.69	0.25	1.20	2.19
Control	all	2.08	0.14	1.79	2.36
Noise 30	1	0.36	0.23	−0.09	0.80
Noise 30	2	1.05	0.28	0.50	1.59
Noise 30	3	0.77	0.26	0.26	1.28
Noise 30	all	0.73	0.15	0.42	1.03
Noise 60	1	0.54	0.26	0.04	1.04
Noise 60	2	0.44	0.20	0.05	0.84
Noise 60	3	0.40	0.20	0.00	0.81
Noise 60	all	0.46	0.14	0.20	0.73

				95% Confidence interval	
Comparison		Mean difference	*P-*value	Lower limit	Upper limit
Control—Noise 30	1	2.76	**<0.001**	2.13	3.39
Control—Noise 30	2	0.37	0.279	−0.30	1.05
Control—Noise 30	3	0.92	**0**.**009**	0.23	1.61
Control—Noise 30	all	1.35	**<0.001**	1.00	1.70
Control—Noise 60	1	2.57	**<0.001**	1.90	3.25
Control—Noise 60	2	0.98	**<0.001**	0.40	1.55
Control—Noise 60	3	1.29	**<0.001**	0.67	1.91
Control—Noise 60	all	1.61	**<0.001**	1.30	1.93
Noise 30—Noise 60	1	−0.18	0.583	−0.84	0.47
Noise 30—Noise 60	2	0.60	0.071	−0.05	1.26
Noise 30—Noise 60	3	0.37	0.254	−0.26	1.00
Noise 30—Noise 60	all	0.26	0.119	−0.07	0.59

Estimates were derived from a linear mixed model with subjects as random effects and exposure (i.e. control scenario, 30 road traffic noise events, and 60 road traffic noise events) and period as fixed effects. Period refers to the chronological order of the study nights (period 1 = first night, period 2 = second night, period 3 = third night), independent of the exposure assignment. Thirty-seven participants had evaluable data for the control night, 44 for the 30-event night, and 39 for the 60-event night. Statistically significant *P-*values (i.e. <0.05) are presented in bold.

### 3.6 Effects of noise on the plasma proteome

**Figure 6:**
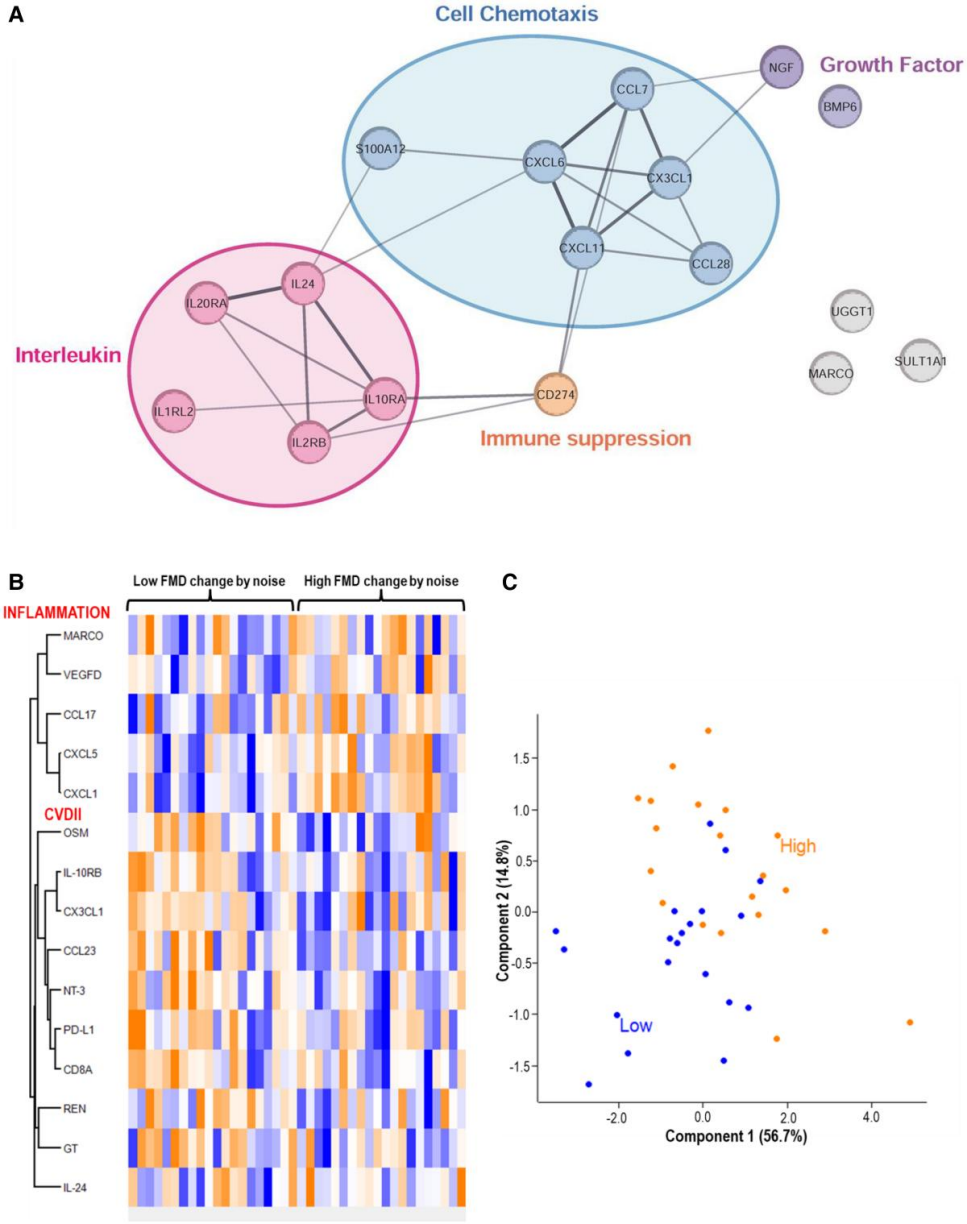
Noise-regulated protein network—total or subgroup analysis. (*A*) Protein interaction network showing proteins with expression changes (*P* < 0.1) before and after noise exposure in a pairwise comparison. Nodes represent proteins, and edges indicate significant interactions, generated using STRING. All samples of both Olink panels (INFLAMMATION and CVDII) were used for combined analysis. (*B*) Noise-induced protein changes in strong and weak FMD responders envisaged by heat map of *z*-normalized protein fold-changes significantly altered between strong and weak FMD responders, defined by the magnitude of noise-induced FMD changes. (*C*) Principal component analysis (PCA) of protein fold-changes. Each dot represents a subject, dark dots (low FMD responders) or bright dots (high FMD responders). A total of 40 subjects were analysed, separated into two groups: the 20 subjects with the lowest FMD changes due to noise and the 20 subjects with the highest FMD changes due to noise. Significance (*P* < 0.05) was determined using an unpaired Student's *t*-test to compare the two groups (strong vs. weak FMD responders). Both Olink panels (INFLAMMATION and CVDII) were used for combined analysis.

When all plasma proteomic data were analysed over all subjects, a noise-regulated protein network centred on IL signaling and cell chemotaxis was found with loose associations to growth factor processes and immune suppression (*Figure 6A*). Few proteins were significantly changed or showed at least a trend (see violin plots, Supplementary material online, *Figure S1*).

When subjects were classified by the extent of noise-induced FMD changes and the 20 subjects with the most pronounced noise-induced FMD changes were compared with those 20 with the smallest FMD reaction to noise, we identified a set of differentially regulated proteins in both Olink panels as envisaged by the heat map (*Figure 6B, C*). Also, the sample distribution showed two distinct patterns for the group with high vs. low FMD changes by noise.

## 4. Discussion

**Figure 7:**
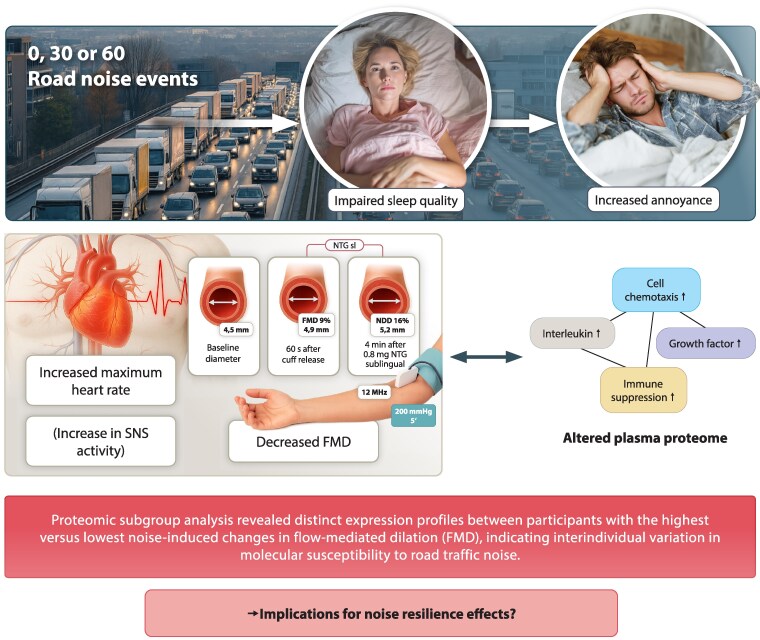
Summary of the randomized, double-blind crossover study exposing healthy adults to 0, 30, or 60 night-time road-traffic-noise events. Road noise exposure impaired sleep quality and increased subjective noise annoyance, reflecting activation of cognitive-emotional stress pathways. Physiologically, noise provoked acute autonomic arousal, demonstrated by an increase in maximum heart rate and elevated sympathetic nervous system (SNS) activity. Endothelial function, assessed by flow-mediated dilation (FMD), was significantly reduced after both 30 and 60 noise events, indicating acute vascular dysfunction despite relatively low indoor sound pressure levels (<45 dB LAeq). Proteomic analyses revealed noise-associated alterations in plasma proteins involved in IL signaling, chemotaxis, immune suppression, and growth-factor pathways. Subgroup comparison of individuals with the highest vs. lowest noise-induced FMD impairment showed distinct proteomic patterns, highlighting interindividual differences in biological susceptibility to noise and pointing towards potential ‘noise resilience’ phenotypes.

This randomized, double-blind, crossover study demonstrates that acute exposure to night-time road traffic noise, even below the average sound pressure levels used previously in our human field studies,^9,12^ induces endothelial dysfunction, increases autonomic cardiovascular activation, and impairs subjective sleep quality in a partly dose-dependent manner in a well-characterized sample of healthy young adults (*Figure 7*). In addition, exploratory proteomic analyses suggest molecular alterations associated with immune signaling pathways and interindividual differences in biological susceptibility to noise. Importantly, we observed more pronounced effects of noise on plasma proteome changes in the subjects with the highest impairment of FMD by noise. These results add mechanistic evidence to epidemiological associations between transportation noise exposure and cardiovascular outcomes and extend previous findings from aircraft and train noise studies to the most ubiquitous source of environmental noise in Europe, being road traffic noise exposure.

The observed reduction in FMD following both 30- and 60-event road traffic noise exposure confirms the sensitivity of endothelial function to short-term environmental noise exposure. These findings are consistent with earlier studies involving recorded aircraft^9–11^ or train noise,^12^ where comparable impairment of FMD was observed.

Increased night-time heart rate under the road traffic noise conditions reflects acute autonomic activation in response to repeated noise events. Heart rate peaks occurred more frequently after incidental noise peaks in control nights than after the standardized events in the 30- and 60-event exposures. This was consistent with the larger post-peak heart rate increases observed during control nights. Intermittent noise may induce stronger autonomic responses than repeated, time-structured events.^24^ The smaller effects in the second and third study nights may further suggest a degree of habituation to the study procedures. These changes were not mirrored in morning blood pressure, consistent with earlier studies showing that noise-induced autonomic responses may be transient and subclinical in the short term.^10^ Despite signs of acute cardiovascular activation (increase in heart rate), circulating levels of adrenaline, IL-6, cortisol, glucose, CRP, and neutrophil granulocytes did not show significant changes across study nights. The lack of measurable alterations may be due to the rather low LAeq values used in the present study, which are lower than those applied previously in our human field studies.^9,12^ Nevertheless, these levels for *L*_night_ (night-time aircraft noise) have been just recently demonstrated to cause structural changes of the heart, such as higher left ventricular mass and also worse LV dynamics within a short time frame.^15^

In contrast, inflammatory and metabolic markers like IL-6, CRP, glucose, and neutrophils may require more sustained or repeated exposure to be affected, particularly in young and healthy individuals. It is also possible that biological responses to noise occurred primarily at the local tissue level without manifesting as systemic changes in peripheral blood. Nonetheless, repeated night-time activation may contribute cumulatively to the pathogenesis of arterial hypertension, inflammation, and atherosclerosis over time. The findings support the role of the sympathetic nervous system as a central mediator in the biological response to environmental noise, even in the absence of conscious arousal.^8,25^

Road traffic noise exposure was associated with consistent and significant impairments across multiple dimensions of self-reported sleep quality. These results are in accordance with earlier studies and confirm that transportation noise disrupts sleep perception^26^ and increases annoyance,^27^ even in young, healthy adults. Importantly, transportation noise annoyance per se has been shown to be associated with incident sleep disturbances^28^ and cardiovascular outcomes^29^ in large cohort studies of the general population. However, subjective impairments were not always proportional to physiological outcomes. This dissociation may reflect variation in individual noise sensitivity, perception thresholds, or cognitive–emotional processing of environmental stress. Importantly, noise annoyance and disturbed sleep are not merely psychological effects suggestive of mental distress but are increasingly recognized as contributors to allostatic load and systemic dysregulation,^30^ with implications for long-term cardiovascular risk.^31^ However, findings from a recent study in the HELIX cohort, which included 919 children aged 6–11 years across six European countries, did not show an association between road traffic noise exposure and allostatic load, highlighting some inconsistencies.^32^

Although only a few group-level differences in conventional biomarkers were detected, stratified analyses revealed distinct proteomic signatures in participants with strong endothelial responses to road traffic noise compared with those showing minor FMD changes. These findings support the concept that interindividual biological susceptibility influences vascular responses to environmental stressors. They also suggest that vulnerability to noise is heterogeneous, helping to identify subgroups who may benefit most from targeted mitigation strategies. Enrichment of immune-related pathways—including IL signaling and chemotaxis—aligns with prior work implicating vascular inflammation in noise-induced vascular dysfunction. Regulation of several IL receptors confirms immunomodulatory effects of noise previously reported in animals.^33,34^ Altered IL-24 expression, which is linked to macrophage migration inhibitory factor–dependent glucocorticoid regulation and TGF-β signaling, further points to modulation of stress hormone responses, tissue repair, and immune-cell activity. Consistent with this, IL-24 is involved in HPA-axis regulation, cortisol reactivity, and anxiety.^35^ TGF-β signaling may proceed through non-canonical routes, including ERK, Ras/Rho GTPase, p38 MAPK, JNK, NF-κB, PI3 K/AKT, and JAK/STAT pathways.^36^ Additional proteins associated with immune activation also envisaged by multiple regulated chemokines. Other important regulated targets comprised PARP1 that is central to apoptosis and DNA repair, transferrin that regulates iron homeostasis, and thrombopoietin that controls platelet production.

Comparative analyses between participants with high vs. low FMD changes revealed additional differentially expressed proteins involved in angiogenesis, T-reg recruitment, neutrophil chemotaxis, monocyte/T-cell chemoattraction, and blood-pressure regulation. Collectively, these proteomic patterns demonstrate that road traffic noise induces heterogeneous immunomodulatory, stress-related, and vascular responses. Importantly, these signatures correlate with individual variability in FMD responses, linking immune and stress-related biology to noise-induced vascular impairment. The latter observation is also compatible with landmark human studies linking circulating markers of inflammation with cardiovascular risk (all coronary heart disease and non-fatal MI or coronary heart disease death)^37^ or impaired FMD.^38–40^ The full discussion of all regulated targets can be found in the Extended Discussion in the online supplement.

In the current study, vitamin C administration did not significantly improve FMD across any exposure condition. While this lack of statistical significance precludes definitive conclusions, a numerically favourable trend was observed in the 60-event noise exposure condition, suggesting a potential, albeit inconclusive, vascular benefit. A similar response under control conditions may indicate a general vasoprotective effect of vitamin C rather than a noise-specific mechanism. The absence of a significant effect may also reflect suboptimal timing, insufficient dosage, or the dominance of non-oxidative mechanisms such as inflammatory or neurohormonal activation in acute road traffic noise responses. Importantly, the subgroup analysis was likely underpowered, with only 31 participants receiving vitamin C, limiting the ability to detect small to moderate effects. High interindividual variability in vascular response and the lack of stratification by noise susceptibility may have further diluted a potentially relevant antioxidant benefit. Nonetheless, these observations are not inconsistent with previous experimental and clinical findings implicating oxidative stress in the cardiovascular effects of noise exposure.^41–43^ Although no direct oxidative stress biomarkers were assessed in this study, the observed pattern aligns with prior work highlighting the role of redox imbalance in noise-induced vascular dysfunction.^9,12^ Future studies with a more rigorous design and biochemical endpoints are warranted to more conclusively test the antioxidant hypothesis in this context. This study has several notable strengths. Unlike prior studies focusing primarily on aircraft or train noise, this study addresses road traffic noise in such a setting for the first time. A rigorous randomized, crossover design was combined with robust statistical modelling, allowing within-subject comparisons while controlling for confounding. The study endpoints were multi-dimensional, including endothelial function, heart rate, subjective sleep parameters, and proteomics, providing a comprehensive view of the acute biological and perceptual effects of night-time road traffic noise. The integration of vascular and molecular endpoints is a particular strength, offering insight into early physiological responses and individual variability.

However, several limitations must also be considered. The study sample consisted of young, healthy participants, which may limit generalizability to older or comorbid populations. While the acute study design allows for assessing short-term effects, it does not account for the cumulative impact of long-term exposure, thereby highlighting the need to consider additional processes such as habituation and physiological adaptation. The sample size for proteomic analyses was relatively small, and findings should be regarded as exploratory. Additionally, the absence of direct oxidative stress markers limits mechanistic interpretation related to redox pathways. While conducting the noise exposure in participants’ homes increases ecological validity, a further potential limitation is the presence of uncontrolled background noise, which may have blurred the effects of the experimental noise conditions. At the same time, one may speculate that noise exposure at home is perceived as more stressful than in a laboratory setting, as it occurs in a personal environment and is not known to be temporary. This could imply that the true effects of night-time road traffic noise may even be more potent than observed in this study. Finally, the observed rather moderate proteomic changes may be a result of the study design using noise exposure for only one night, which may prevent pronounced regulation of protein targets that need induction of mRNA and translation to protein expression.

In conclusion, the present study provides human experimental evidence that acute night-time exposure to road traffic noise impairs endothelial function, elevates heart rate, and disrupts subjective sleep quality in young and otherwise healthy adults. These effects were already observed after a single night of road traffic noise exposure, using noise levels and patterns that reflect typical urban environments. The vascular and autonomic changes detected are consistent with mechanisms implicated in the early development of cardiovascular disease. Given that endothelial dysfunction is an established precursor to atherosclerosis and predictive of future cardiovascular events,^44^ these findings carry direct clinical relevance. This highlights the need for precise preventive strategies that address environmental determinants of cardiovascular health. Future studies should also assess the potential role of earplugs, which can reduce perceived noise and night-time disturbance, although evidence for preventing cardiovascular effects is currently lacking and inconclusive.^45^ From a public health perspective, the findings strengthen the case for stricter regulation of night-time noise levels in residential areas, especially in urban settings with high traffic density. Considering that the here employed LAeq values for acute exposure to night-time road traffic noise were lower than those used previously,^9,12^ the observed functional and biochemical changes are striking and underline the need for further studies on chronic noise exposure close to the recommended WHO thresholds. From a translational perspective, noise-mitigation strategies should therefore combine population-level measures, such as stricter night-time noise limits and urban planning interventions, with personal approaches, including ear protection, sleep-related behavioural strategies, and targeted cardiovascular risk assessment, particularly in individuals with higher exposure or suspected susceptibility.

Translational perspective This randomized, double-blind, crossover study shows that even low-level night-time road traffic noise (LAeq <45 dB) acutely impairs endothelial function, elevates heart rate, disrupts sleep, and alters plasma protein expression in healthy adults. Proteomic signatures point to immune activation and stress response pathways, highlighting molecular mechanisms underlying noise-induced vascular damage. These findings strengthen the evidence that environmental noise is not just a nuisance but a modifiable cardiovascular risk factor. They support calls by the World Health Organization (WHO) and European Society of Cardiology (ESC) for stricter noise regulation and underscore the need for urban planning that protects sleep and vascular health in increasingly noise-polluted environments.

## Supplementary Material

cvag028_Supplementary_Data

## Data Availability

Data are all contained within this article. Raw data are available from the corresponding author upon reasonable request.
