# Supplementary material for: A randomized, double-blind, crossover study of acute low-level night-time road traffic noise: effects on vascular function, sleep, and proteomic signatures in healthy adults
Source: Cardiovasc Res. 2026 Feb 25;122(5):621–36. doi: 10.1093/cvr/cvag028 (PMC13020542; doi:10.1093/cvr/cvag028)
Supplement: cvag028_Supplementary_Data [file cvag028_supplementary_data.docx]

**Table S1.** Effects of nighttime road traffic noise exposure on the primary endpoint flow-mediated dilation (FMD) (N=219^+^).

| **A)** | **Exposure** | **Mean** | **Standard error** | **95% confidence interval** |
| --- | --- | --- | --- | --- |
| FMD (%) | Control | 9.35 | 0.38 | 8.61; 10.10 |
|  | Noise 30 | 8.19 | 0.38 | 7.44; 8.95 |
|  | Noise 60 | 7.73 | 0.38 | 6.97; 8.48 |

| **B)** | **Exposure comparison** | **Mean difference** | **Standard error** | **P value** | **95% confidence interval** |
| --- | --- | --- | --- | --- | --- |
| FMD (%) | Control-noise 30 | 1.16 | 0.41 | **0.005** | 0.36; 1.96 |
|  | Control-noise 60 | 1.63 | 0.41 | **<0.0001** | 0.82; 2.43 |
|  | Noise 30-noise 60 | 0.47 | 0.41 | 0.256 | -0.34; 1.28 |

^+^ All participants contributed measurements for Control, for 2 there was no measurement at Noise 30, for 1 there was no measurement at Noise 60.

Estimates were derived from a mixed linear model with subjects as a random effect and exposure (i.e. control scenario, 30 road traffic noise events, and 60 road traffic noise events) as a fixed effect. A) refers to the model-based estimates. B) refers to the mean differences and pairwise comparisons. Statistically significant p values (i.e. <0.05) are presented in bold.

**Table S2.** Flow-mediated dilation (FMD) before and 2 hours after vitamin C intake across exposure conditions and comparison of changes with vs. without vitamin C (N=218*).

| 1. **Exposure** | **Vitamin C** | **Mean FMD before (%)** | **SD** | **Mean FMD 2h after (%)** | **SD** | **Mean ΔFMD (%)** | **SD** |
| --- | --- | --- | --- | --- | --- | --- | --- |
| Control | No | 9.52 | 3.32 | 11.77 | 3.50 | 2.23 | 1.23 |
|  | Yes | 9.11 | 3.69 | 11.87 | 4.37 | 2.76 | 2.06 |
| Noise 30 | No | 8.81 | 2.57 | 12.10 | 3.78 | 3.29 | 3.20 |
|  | Yes | 7.57 | 3.19 | 10.86 | 4.68 | 3.29 | 3.33 |
| Noise 60 | No | 8.13 | 3.29 | 11.23 | 3.23 | 3.10 | 2.02 |
|  | Yes | 7.22 | 2.96 | 11.34 | 3.50 | 4.12 | 2.74 |
|  |  |  |  |  |  |  |  |

| **B)** | **Exposure comparison** | **Mean difference** | **Standard error** | **P value** | **95% confidence interval** |
| --- | --- | --- | --- | --- | --- |
| Difference in change of FMD (%) between vitamin C and no vitamin C intake | Control | 0.52 | 0.59 | 0.38 | -0.65; 1.69 |
|  | Noise 30 | 0.01 | 0.60 | 0.99 | -1.16; 1.18 |
|  | Noise 60 | 1.02 | 0.59 | 0.09 | -0.15; 2.20 |

* 31 participants received vitamin C and contributed measurement at all exposures, 43 participants served as controls, for 2 there was no measurement at Noise 30, for 1 there was no measurement at Noise 60, for 1 the measurement after 2 hours was missing at control.

A) Absolute FMD values before and 2 hours after vitamin C/no vitamin C intake and corresponding ΔFMD values across exposure conditions (control, 30 events, 60 events). Values represent descriptive statistics (mean and standard deviation (SD)). ΔFMD is defined as the difference between the FMD measurement 2 hours after vitamin C/no vitamin C and the baseline FMD measurement.

B) Model-based differences in ΔFMD between vitamin C and no vitamin C intake. Estimates were derived from a linear mixed model including exposure (control, 30 events, 60 events) and vitamin C intake (yes/no) as fixed effects and participant as a random effect. Mean difference represents the difference in ΔFMD between vitamin C and no vitamin C. The interval between baseline and the second FMD measurement was 2 hours.

|  |  | |  | | |  | | **Table S3.** Associations of nighttime road traffic noise exposure on secondary endpoints. | | | | | | | | | | |
| --- | --- | --- | --- | --- | --- | --- | --- | --- | --- | --- | --- | --- | --- | --- | --- | --- | --- | --- |
| **Variable** | | **Exposure** | | **N** | **Mean** | | **Standard deviation** | | **Min** | **Q1** | **Median** | **Q3** | **Max** | **Exposure comparison** | **Mean difference** | **Standard error** | **P value** | **95% confidence interval** |
|  |  | |  | | |  | | *Noise exposure/annoyance* | | | | | | | | | | |
| Peak sound level (dB) | | Control | | 72 | 58.39 | | 8.39 | | 44.10 | 52.10 | 57.00 | 65.15 | 79.20 | Control-noise 30 | -4.03 | 1.09 | **0.0003** | -6.19; -1.86 |
|  |  | Noise 30 | | 69 | 62.11 | | 6.38 | | 54.40 | 58.40 | 60.40 | 63.10 | 90.20 | Control-noise 60 | -2.95 | 1.09 | **0.01** | -5.09; -0.80 |
|  |  | Noise 60 | | 71 | 61.00 | | 5.84 | | 31.60 | 58.40 | 59.40 | 63.20 | 76.80 | Noise 30-noise 60 | 1.08 | 1.10 | 0.33 | -1.10;3.26 |
| A-weighted equivalent continuous sound levels (LAeq in dB) | | Control | | 72 | 30.70 | | 3.67 | | 25.10 | 28.01 | 29.90 | 32.38 | 43.92 | Control-noise 30 | -10.95 | 0.50 | **< 0.0001** | (-11.93; -9.97) |
|  |  | Noise 30 | | 69 | 41.36 | | 2.82 | | 34.49 | 39.49 | 41.16 | 43.24 | 50.19 | Control-noise 60 | -13.51 | 0.49 | **< 0.0001** | -14.48; -12.53 |
|  |  | Noise 60 | | 71 | 44.13 | | 3.42 | | 38.03 | 42.87 | 44.24 | 45.21 | 64.41 | Noise 30-noise 60 | -2.56 | 0.50 | **< 0.0001** | -3.55; -1.57 |
| "How annoyed were you by road traffic noise?" (1 (not at all) to 5 (extremely) points | | Control | | 74 | 1.77 | | 0.91 | | 1.00 | 1.00 | 2.00 | 2.00 | 4.00 | Control-noise 30 | -1.18 | 0.13 | **<.0001** | -1.45, -0.92 |
|  |  | Noise 30 | | 74 | 2.96 | | 1.08 | | 1.00 | 2.00 | 3.00 | 4.00 | 5.00 | Control-noise 60 | -1.31 | 0.13 | **<.0001** | -1.58; -1.05 |
|  |  | Noise 60 | | 74 | 3.15 | | 1.04 | | 1.00 | 2.00 | 3.00 | 4.00 | 5.00 | Noise 30-noise 60 | -0.13 | 0.13 | 0.34 | -0.40; 0.14 |
|  |  | |  | | |  | | *Hemodynamic* | | | | | | | | | | |
| Systolic blood pressure before FMD measurement (mmHg) | | Control | | 74 | 123.59 | | 9.42 | | 105.00 | 116.00 | 122.00 | 131.00 | 153.00 | Control-noise 30 | -0.49 | 1.05 | 0.64 | -2.57;1.59 |
|  |  | Noise 30 | | 74 | 124.00 | | 9.67 | | 105.00 | 117.00 | 123.00 | 131.00 | 148.00 | Control-noise 60 | -1.02 | 1.06 | 0.34 | -3.11;1.08 |
|  |  | Noise 60 | | 74 | 124.22 | | 11.21 | | 104.00 | 116.00 | 124.00 | 132.00 | 161.00 | Noise 30-noise 60 | -0.52 | 1.06 | 0.62 | -2.62;1.57 |
| Diastolic blood pressure before FMD measurement (mmHg) | | Control | | 74 | 73.99 | | 8.48 | | 42.00 | 69.00 | 74.00 | 78.00 | 98.00 | Control-noise 30 | -0.52 | 0.80 | 0.51 | -2.11;1.06 |
|  |  | Noise 30 | | 74 | 74.54 | | 7.25 | | 56.00 | 70.00 | 74.00 | 79.00 | 95.00 | Control-noise 60 | -0.33 | 0.81 | 0.68 | -1.92;1.26 |
|  |  | Noise 60 | | 74 | 74.19 | | 7.23 | | 57.00 | 70.00 | 73.00 | 77.00 | 97.00 | Noise 30-noise 60 | 0.19 | 0.81 | 0.81 | -1.40;1.79 |
| Min. pulse transit time (m/s) | | Control | | 72 | 291.07 | | 40.48 | | 0.00 | 279.00 | 294.00 | 307.50 | 355.00 | Control-noise 30 | -3.88 | 3.81 | 0.31 | -11.42;3.67 |
|  |  | Noise 30 | | 72 | 294.63 | | 16.77 | | 260.00 | 286.50 | 293.00 | 301.00 | 337.00 | Control-noise 60 | -5.98 | 3.82 | 0.12 | -13.54;1.59 |
|  |  | Noise 60 | | 73 | 296.79 | | 21.14 | | 257.00 | 283.00 | 292.00 | 310.00 | 390.00 | Noise 30-noise 60 | -2.10 | 3.82 | 0.58 | -9.66;5.46 |
| Mean pulse transit time (m/s) | | Control | | 72 | 335.94 | | 44.20 | | 0.00 | 328.50 | 339.00 | 350.50 | 379.00 | Control-noise 30 | -5.11 | 4.05 | 0.21 | -13.12;2.91 |
|  |  | Noise 30 | | 72 | 340.26 | | 17.28 | | 303.00 | 330.50 | 340.50 | 346.50 | 384.00 | Control-noise 60 | -5.10 | 4.06 | 0.21 | -13.13;2.94 |
|  |  | Noise 60 | | 73 | 340.70 | | 18.99 | | 300.00 | 327.00 | 340.00 | 355.00 | 382.00 | Noise 30-noise 60 | 0.01 | 4.06 | 1.00 | -8.02;8.05 |
| Max. pulse transit time (m/s) | | Control | | 72 | 383.49 | | 56.14 | | 0.00 | 361.50 | 386.00 | 410.00 | 487.00 | Control-noise 30 | -3.47 | 6.13 | 0.57 | -15.60;8.66 |
|  |  | Noise 30 | | 72 | 385.51 | | 30.22 | | 329.00 | 366.00 | 382.50 | 402.00 | 489.00 | Control-noise 60 | -1.06 | 6.15 | 0.86 | -13.21;11.10 |
|  |  | Noise 60 | | 73 | 384.29 | | 27.44 | | 329.00 | 364.00 | 382.00 | 402.00 | 476.00 | Noise 30-noise 60 | 2.41 | 6.15 | 0.70 | -9.75;14.57 |
| Systolic blood pressure during study night (mmHg) | | Control | | 71 | 121.72 | | 14.97 | | 92.00 | 110.00 | 120.00 | 132.00 | 170.00 | Control-noise 30 | -0.30 | 1.97 | 0.88 | -4.20;3.59 |
|  |  | Noise 30 | | 72 | 122.63 | | 14.19 | | 90.00 | 113.50 | 121.50 | 130.50 | 172.00 | Control-noise 60 | 1.21 | 1.98 | 0.54 | -2.69;5.12 |
|  |  | Noise 60 | | 73 | 120.92 | | 15.63 | | 62.00 | 112.00 | 120.00 | 129.00 | 154.00 | Noise 30-noise 60 | 1.52 | 1.96 | 0.44 | -2.36;5.40 |
| Diastolic blood pressure during study night (mmHg) | | Control | | 71 | 78.07 | | 11.39 | | 48.00 | 71.00 | 76.00 | 85.00 | 117.00 | Control-noise 30 | -0.66 | 1.60 | 0.68 | -3.81;2.50 |
|  |  | Noise 30 | | 72 | 78.67 | | 11.93 | | 44.00 | 71.50 | 79.50 | 87.00 | 115.00 | Control-noise 60 | 0.91 | 1.60 | 0.57 | -2.25;4.08 |
|  |  | Noise 60 | | 73 | 76.81 | | 10.10 | | 55.00 | 70.00 | 77.00 | 83.00 | 101.00 | Noise 30-noise 60 | 1.57 | 1.59 | 0.32 | -1.57;4.72 |
| Acceleration of blood pressure during study night (1/h) | | Control | | 71 | 5.68 | | 5.80 | | 0.00 | 2.70 | 3.90 | 6.30 | 34.30 | Control-noise 30 | -0.42 | 0.64 | 0.52 | -1.69;0.86 |
|  |  | Noise 30 | | 72 | 6.06 | | 7.61 | | 0.30 | 2.45 | 4.15 | 6.55 | 49.00 | Control-noise 60 | -1.03 | 0.65 | 0.11 | -2.30;0.25 |
|  |  | Noise 60 | | 73 | 6.39 | | 6.70 | | 0.20 | 2.60 | 5.00 | 7.90 | 45.90 | Noise 30-noise 60 | -0.61 | 0.64 | 0.35 | -1.88;0.66 |
| Heart rate (bpm) | | Control | | 73 | 59.49 | | 7.34 | | 42.00 | 54.00 | 59.00 | 64.00 | 80.00 | Control-noise 30 | 0.17 | 0.58 | 0.76 | -0.97;1.32 |
|  |  | Noise 30 | | 73 | 59.37 | | 7.36 | | 40.00 | 55.00 | 60.00 | 65.00 | 74.00 | Control-noise 60 | -1.23 | 0.58 | **0.04** | -2.37; -0.08 |
|  |  | Noise 60 | | 74 | 60.77 | | 7.38 | | 44.00 | 55.00 | 62.00 | 66.00 | 76.00 | Noise 30-noise 60 | -1.40 | 0.58 | **0.02** | -2.55; -0.25 |
| Max. heart rate (bpm) | | Control | | 73 | 99.84 | | 13.18 | | 52.00 | 93.00 | 100.00 | 106.00 | 139.00 | Control-noise 30 | -0.57 | 2.15 | 0.79 | -4.83;3.69 |
|  |  | Noise 30 | | 73 | 100.38 | | 12.65 | | 54.00 | 93.00 | 99.00 | 108.00 | 142.00 | Control-noise 60 | -7.95 | 2.16 | **<0.001** | -12.22; -3.67 |
|  |  | Noise 60 | | 74 | 107.24 | | 19.45 | | 77.00 | 96.00 | 103.00 | 113.00 | 180.00 | Noise 30-noise 60 | -7.38 | 2.16 | **<0.001** | -11.65;-3.10) |
| Acceleration of Heart rate (1/h) | | Control | | 73 | 17.87 | | 16.52 | | 2.00 | 7.50 | 13.10 | 22.40 | 100.40 | Control-noise 30 | -1.94 | 1.38 | 0.16 | -4.68;0.80 |
|  |  | Noise 30 | | 73 | 19.83 | | 23.17 | | 0.00 | 6.70 | 12.80 | 23.50 | 149.20 | Control-noise 60 | -0.94 | 1.39 | 0.50 | -3.69;1.80 |
|  |  | Noise 60 | | 74 | 18.93 | | 18.36 | | 2.80 | 8.10 | 13.15 | 23.20 | 113.20 | Noise 30-noise 60 | 1.00 | 1.39 | 0.47 | -1.75;3.74 |
|  |  | |  | | |  | | *Sleep quality* | | | | | | | | | | |
| “How do you feel right now” evening (0 (very good) to 15 (very bad) points) | | Control | | 74 | 5.74 | | 2.81 | | 0.00 | 4.00 | 6.00 | 7.00 | 12.00 | Control-noise 30 | 0.59 | 0.36 | 0.10 | -0.11; -1.30 |
|  |  | Noise 30 | | 74 | 5.28 | | 2.68 | | 0.00 | 4.00 | 5.00 | 7.00 | 12.00 | Control-noise 60 | 0.10 | 0.36 | 0.79 | -0.61; 0.81 |
|  |  | Noise 60 | | 74 | 5.65 | | 2.77 | | 0.00 | 3.00 | 5.00 | 8.00 | 13.00 | Noise 30-noise 60 | -0.50 | 0.36 | 0.17 | -1.21;0.21 |
| “How do you feel right now” morning (0 (very good) to 15 (very bad) points) | | Control | | 74 | 5.72 | | 2.60 | | 1.00 | 4.00 | 6.00 | 7.00 | 15.00 | Control-noise 30 | -0.88 | 0.36 | **0.02** | -1.59;-0.17 |
|  |  | Noise 30 | | 74 | 6.64 | | 2.67 | | 0.00 | 5.00 | 7.00 | 9.00 | 12.00 | Control-noise 60 | -1.46 | 0.36 | **<0.0001** | -2.17;-0.75 |
|  |  | Noise 60 | | 74 | 7.12 | | 2.56 | | 2.00 | 5.00 | 7.00 | 9.00 | 13.00 | Noise 30-noise 60 | -0.58 | 0.36 | 0.11 | -1.30;0.13 |
| “Sleep quality overall” VAS (0 (very good) to 10 (very bad) points) | | Control | | 74 | 3.70 | | 2.15 | | 0.20 | 2.10 | 3.00 | 5.70 | 8.50 | Control-noise 30 | -1.77 | 0.30 | **<0.0001** | -2.37;-1.16 |
|  |  | Noise 30 | | 74 | 5.47 | | 2.36 | | 0.70 | 3.70 | 5.75 | 7.20 | 10.00 | Control-noise 60 | -2.33 | 0.31 | **<0.0001** | -2.94;-1.72 |
|  |  | Noise 60 | | 74 | 5.99 | | 2.11 | | 0.30 | 4.60 | 6.00 | 7.50 | 10.00 | Noise 30-noise 60 | -0.56 | 0.31 | 0.07 | -1.17;0.04 |
| “How restful was your sleep” (0 (extremely) to 4 (not at all) points) | | Control | | 74 | 1.46 | | 0.78 | | 0.00 | 1.00 | 1.50 | 2.00 | 3.00 | Control-noise 30 | -0.57 | 0.11 | **<.0001** | -0.80; -0.35 |
|  |  | Noise 30 | | 73 | 2.01 | | 0.96 | | 0.00 | 1.00 | 2.00 | 3.00 | 4.00 | Control-noise 60 | -0.86 | 0.11 | **<.0001** | -1.08; -0.64 |
|  |  | Noise 60 | | 74 | 2.28 | | 0.84 | | 0.00 | 2.00 | 2.00 | 3.00 | 4.00 | Noise 30-noise 60 | -0.29 | 0.11 | **0.01** | -0.51; - 0.06 |
| Sleep quality scale (0 (happy) to 10 (unhappy) points) | | Control | | 73 | 2.79 | | 1.71 | | 0.00 | 2.00 | 2.00 | 3.00 | 9.00 | Control-noise 30 | -1.15 | 0.24 | **<.0001** | -1.63;-0.67 |
|  |  | Noise 30 | | 74 | 3.99 | | 1.83 | | 0.50 | 3.00 | 4.00 | 5.00 | 8.00 | Control-noise 60 | -1.50 | 0.24 | **<.0001** | -1.98;-1.01 |
|  |  | Noise 60 | | 73 | 4.26 | | 1.94 | | 0.00 | 3.00 | 4.00 | 6.00 | 9.00 | Noise 30-noise 60 | -0.35 | 0.24 | 0.16 | -0.83;0.14 |
| Sleep quality scale (0 (fit) to 10 (exhausted) points) | | Control | | 73 | 4.01 | | 2.26 | | 0.00 | 2.00 | 3.00 | 6.00 | 9.40 | Control-noise 30 | -1.44 | 0.30 | **<0.001** | -2.04;-0.84 |
|  |  | Noise 30 | | 74 | 5.44 | | 2.14 | | 1.00 | 4.00 | 6.00 | 7.00 | 10.00 | Control-noise 60 | -1.64 | 0.31 | **<0.0001** | -2.25;-1.04 |
|  |  | Noise 60 | | 73 | 5.61 | | 2.17 | | 1.00 | 4.00 | 6.00 | 7.00 | 10.00 | Noise 30-noise 60 | -0.20 | 0.31 | 0.51 | -0.81;0.40 |
| Sleep quality scale (0 (relaxed) to 10 (stressed) points) | | Control | | 73 | 2.89 | | 1.98 | | 0.00 | 1.00 | 3.00 | 4.00 | 9.00 | Control-noise 30 | -1.12 | 0.26 | **<0.0001** | -1.64;-0.61 |
|  |  | Noise 30 | | 74 | 4.06 | | 2.05 | | 0.00 | 2.00 | 4.00 | 6.00 | 8.00 | Control-noise 60 | -1.50 | 0.26 | **<0.0001** | -2.02;-0.98 |
|  |  | Noise 60 | | 73 | 4.35 | | 2.08 | | 1.00 | 3.00 | 4.00 | 6.00 | 9.00 | Noise 30-noise 60 | -0.38 | 0.26 | 0.16 | -0.90;0.15 |
| "Difficulty falling asleep” (0 (easy) to 10 (difficult) points) | | Control | | 73 | 3.66 | | 2.49 | | 0.00 | 2.00 | 3.00 | 5.00 | 9.00 | Control-noise 30 | -0.86 | 0.36 | **0.02** | -1.56;-0.16 |
|  |  | Noise 30 | | 74 | 4.57 | | 2.64 | | 0.50 | 2.00 | 5.00 | 7.00 | 10.00 | Control-noise 60 | -1.48 | 0.36 | **<0.0001** | -2.19;-0.77 |
|  |  | Noise 60 | | 74 | 5.04 | | 2.94 | | 0.00 | 2.50 | 5.00 | 7.00 | 10.00 | Noise 30-noise 60 | -0.62 | 0.36 | 0.08 | -1.33;0.08 |
| "Sleep was...” (0 (calm) to 10 (restless) points) | | Control | | 73 | 3.85 | | 2.13 | | 0.00 | 2.00 | 4.00 | 5.00 | 9.00 | Control-noise 30 | -1.91 | 0.28 | **<0.0001** | -2.47;-1.35 |
|  |  | Noise 30 | | 74 | 5.78 | | 2.08 | | 1.00 | 4.00 | 6.00 | 7.00 | 10.00 | Control-noise 60 | -2.59 | 0.28 | **<0.0001** | -3.15;-2.03 |
|  |  | Noise 60 | | 74 | 6.41 | | 2.00 | | 1.00 | 5.00 | 7.00 | 8.00 | 10.00 | Noise 30-noise 60 | -0.68 | 0.28 | **0.02** | -1.24;-0.12 |
| "Sleep depth” (0 (deep) to 10 (light) points) | | Control | | 73 | 3.82 | | 2.09 | | 0.00 | 2.00 | 3.00 | 5.00 | 10.00 | Control-noise 30 | -1.20 | 0.30 | **0.0001** | -1.79;-0.60 |
|  |  | Noise 30 | | 74 | 5.03 | | 2.24 | | 1.00 | 3.00 | 5.00 | 7.00 | 10.00 | Control-noise 60 | -1.88 | 0.30 | **<0.0001** | -2.47;-1.28 |
|  |  | Noise 60 | | 74 | 5.64 | | 2.30 | | 1.00 | 4.00 | 6.00 | 7.00 | 10.00 | Noise 30-noise 60 | -0.68 | 0.30 | **0.03** | -1.28;-0.08 |
| "Sleep duration” (0 (short) to 10 (long) points) | | Control | | 73 | 4.71 | | 1.52 | | 2.00 | 4.00 | 5.00 | 5.00 | 9.00 | Control-noise 30 | 0.51 | 0.23 | **0.03** | 0.05;0.97 |
|  |  | Noise 30 | | 74 | 4.23 | | 1.58 | | 0.00 | 3.00 | 4.00 | 5.00 | 8.00 | Control-noise 60 | 0.61 | 0.23 | 0.01 | 0.16;1.07 |
|  |  | Noise 60 | | 74 | 4.12 | | 1.64 | | 0.50 | 3.00 | 4.00 | 5.00 | 8.00 | Noise 30-noise 60 | 0.10 | 0.23 | 0.65 | -0.35;0.56 |
| "Sleep recovery” (0 (high) to 10 points) | | Control | | 73 | 4.01 | | 1.93 | | 0.00 | 3.00 | 3.00 | 6.00 | 9.00 | Control-noise 30 | -1.60 | 0.26 | **<0.0001** | -2.12;-1.09 |
|  |  | Noise 30 | | 74 | 5.68 | | 1.95 | | 1.00 | 4.00 | 6.00 | 7.00 | 9.00 | Control-noise 60 | -2.14 | 0.26 | **<0.0001** | -2.66;-1.62 |
|  |  | Noise 60 | | 74 | 6.12 | | 1.85 | | 1.00 | 5.00 | 6.00 | 7.00 | 10.00 | Noise 30-noise 60 | -0.54 | 0.26 | **0.04** | -1.06;-0.02 |
| "Frequency of movement” (0 (little) to 10 (much) points) | | Control | | 73 | 4.68 | | 2.17 | | 1.00 | 3.00 | 5.00 | 6.00 | 9.00 | Control-noise 30 | -0.59 | 0.30 | **0.05** | -1.19;-0.0002 |
|  |  | Noise 30 | | 74 | 5.28 | | 2.09 | | 0.00 | 4.00 | 6.00 | 6.50 | 10.00 | Control-noise 60 | -1.15 | 0.30 | **0.0002** | -1.75;-0.55 |
|  |  | Noise 60 | | 74 | 5.81 | | 2.14 | | 0.50 | 5.00 | 6.00 | 7.00 | 10.00 | Noise 30-noise 60 | -0.56 | 0.30 | 0.07 | -1.15;0.04 |
| “Sleep quality compared to usual" (1 (very good) to 5 (very poor) points | | Control | | 73 | 3.30 | | 0.66 | | 1.00 | 3.00 | 3.00 | 4.00 | 4.00 | Control-noise 30 | -0.47 | 0.08 | **<.0001** | -0.63; -0.31 |
|  |  | Noise 30 | | 74 | 3.77 | | 0.45 | | 3.00 | 4.00 | 4.00 | 4.00 | 5.00 | Control-noise 60 | -0.54 | 0.08 | **<.0001** | -0.70; -0.38 |
|  |  | Noise 60 | | 74 | 3.84 | | 0.52 | | 1.00 | 4.00 | 4.00 | 4.00 | 5.00 | Noise 30-noise 60 | -0.07 | 0.08 | 0.39 | -0.23; 0.09 |
|  |  | |  | | |  | | *Blood chemistry* | | | | | | | | | | |
| log(Adrenaline) (pg/mL) | | Control | | 60 | 44.03 | | 22.74 | | 20.00 | 20.00 | 41.90 | 57.85 | 102.00 | Control-noise 30 | 4.45 | 3.79 | 0.24 | -3.06; 11.96 |
|  |  | Noise 30 | | 60 | 44.18 | | 19.96 | | 20.00 | 20.00 | 39.20 | 50.35 | 99.50 | Control-noise 60 | 3.17 | 3.83 | 0.41 | -10.77; 4.42 |
|  |  | Noise 60 | | 58 | 47.66 | | 21.76 | | 20.00 | 33.30 | 47.10 | 58.60 | 123.00 | Noise 30-noise 60 | -7.62 | 3.86 | 0.051 | -15.27; 0.02 |
| Neutrophil granulocytes (%) | | Control | | 73 | 53.49 | | 8.80 | | 31.80 | 47.50 | 52.60 | 59.70 | 79.70 | Control-noise 30 | 0.80 | 0.92 | 0.39 | -1.02;2.61 |
|  |  | Noise 30 | | 74 | 52.44 | | 8.30 | | 32.40 | 46.20 | 53.20 | 58.70 | 68.20 | Control-noise 60 | 0.14 | 0.93 | 0.88 | -1.69;1.98 |
|  |  | Noise 60 | | 73 | 53.10 | | 9.28 | | 30.40 | 48.70 | 54.40 | 58.70 | 78.00 | Noise 30-noise 60 | -0.65 | 0.92 | 0.48 | -2.48;1.17 |
| log(Interleukin-6) (ng/L) | | Control | | 74 | 2.85 | | 2.19 | | 2.00 | 2.00 | 2.00 | 3.00 | 19.00 | Control-noise 30 | 0.03 | 0.05 | 0.53 | -0.07;0.13 |
|  |  | Noise 30 | | 74 | 2.80 | | 2.14 | | 2.00 | 2.00 | 2.00 | 3.00 | 18.00 | Control-noise 60 | 0.00 | 0.05 | 0.99 | -0.10;0.10 |
|  |  | Noise 60 | | 74 | 2.85 | | 1.86 | | 2.00 | 2.00 | 2.00 | 3.00 | 13.00 | Noise 30-noise 60 | -0.03 | 0.05 | 0.54 | -0.13;0.07 |
| Cortisol (µg/L) | | Control | | 74 | 14.51 | | 4.90 | | 6.30 | 11.40 | 13.40 | 15.80 | 31.60 | Control-noise 30 | 0.39 | 0.36 | 0.29 | -0.33;1.11 |
|  |  | Noise 30 | | 74 | 14.22 | | 4.69 | | 6.80 | 11.10 | 13.20 | 15.80 | 28.90 | Control-noise 60 | 0.19 | 0.37 | 0.60 | -0.53;0.92 |
|  |  | Noise 60 | | 74 | 14.37 | | 5.01 | | 6.70 | 10.90 | 13.50 | 15.80 | 30.00 | Noise 30-noise 60 | -0.19 | 0.37 | 0.60 | -0.92;0.53 |
| Glucose (mg/dL) | | Control | | 74 | 89.47 | | 7.00 | | 78.00 | 84.00 | 89.50 | 94.00 | 109.00 | Control-noise 30 | 0.33 | 0.74 | 0.65 | -1.13;1.80 |
|  |  | Noise 30 | | 74 | 89.30 | | 7.17 | | 76.00 | 84.00 | 89.00 | 94.00 | 108.00 | Control-noise 60 | 0.21 | 0.74 | 0.77 | -1.26;1.69 |
|  |  | Noise 60 | | 74 | 89.24 | | 8.01 | | 74.00 | 84.00 | 89.50 | 95.00 | 106.00 | Noise 30-noise 60 | -0.12 | 0.75 | 0.87 | -1.59;1.35 |
| Log(C-reactive protein) (mg/L) | | Control | | 74 | 2.08 | | 7.17 | | 0.20 | 0.45 | 0.75 | 1.20 | 61.00 | Control-noise 30 | 0.00 | 0.10 | 0.97 | -0.21;0.20 |
|  |  | Noise 30 | | 74 | 1.14 | | 0.95 | | 0.20 | 0.48 | 0.81 | 1.40 | 4.40 | Control-noise 60 | -0.07 | 0.10 | 0.51 | -0.27;0.14 |
|  |  | Noise 60 | | 73 | 1.77 | | 4.14 | | 0.20 | 0.45 | 0.82 | 1.40 | 31.00 | Noise 30-noise 60 | 0.07 | 0.10 | 0.53 | -0.27;0.14 |

Estimates were derived from a mixed linear model with subjects as a random effect and exposure (i.e. control scenario, 30 road traffic noise events, and 60 road traffic noise events) as a fixed effect. Statistically significant p values (i.e. <0.05) are presented in bold.

**
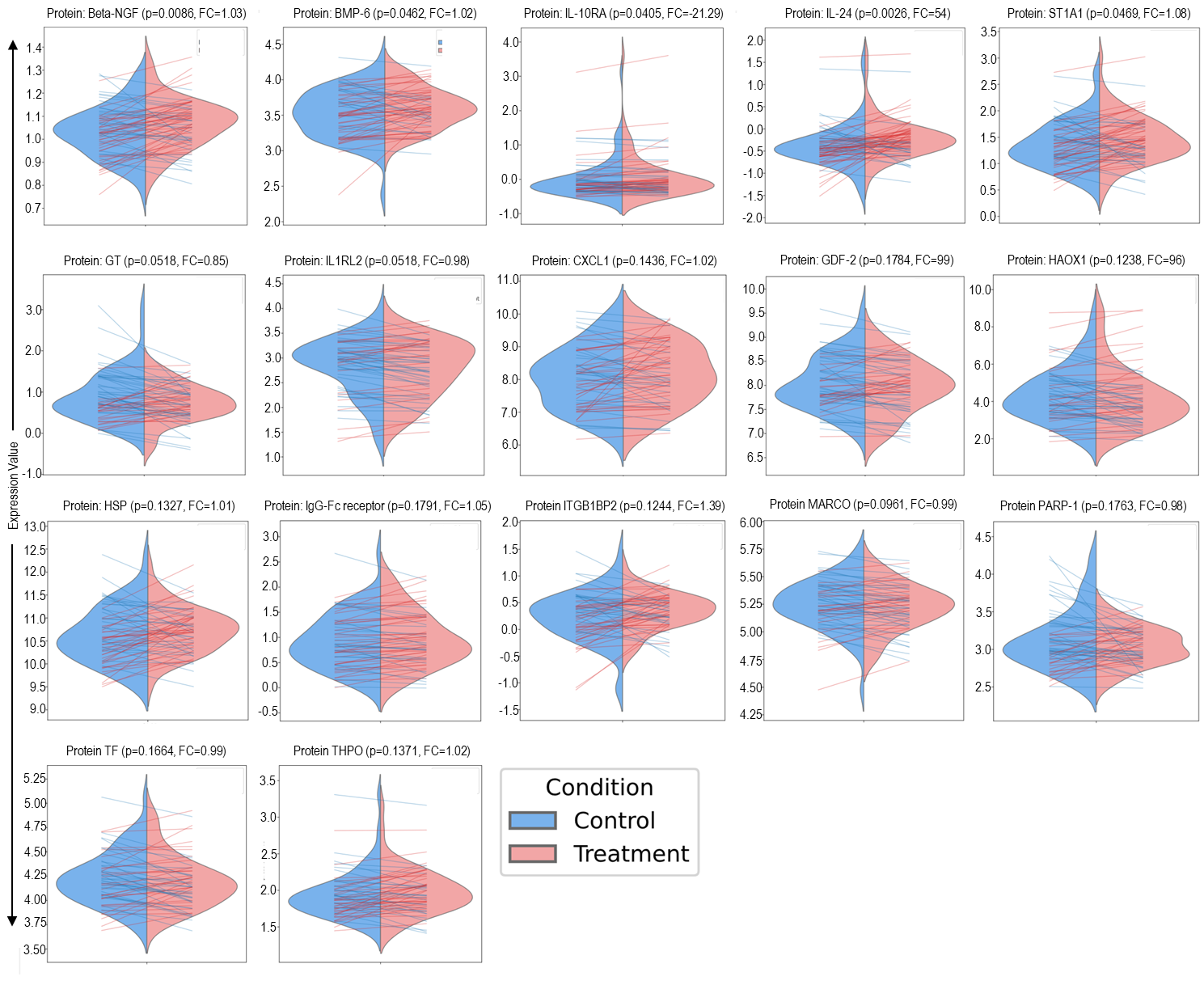
**

**Suppl. Figure S1**. **Noise-regulated protein network.** Violin plots depicting protein expression intensities before (blue) and after (red) noise exposure. Red and blue lines indicate increased or decreased expression, respectively, in individual subjects. Proteins were included based on a significance threshold of p < 0.2 to capture stable regulatory trends. Sixty-one paired samples were analysed. For each pair, protein expression changes were compared between Control and Noise60 conditions. The P-values were calculated using a paired t-test as implemented in the SciPy library in Python. All samples of both Olink panels (INFLAMMATION and CVDII) were used for combined analysis.

**Extended Discussion of Changes of the Plasma Proteome**

Although only few group-level differences in conventional biomarkers were found, stratified analyses revealed distinct proteomic profiles in participants with stronger endothelial responses to road traffic noise as compared to those with minor changes of FMD in response to noise. These findings support the notion that interindividual biological susceptibility plays a role in shaping vascular responses to environmental stressors. These observations suggest that biological vulnerability to environmental noise is not uniform across individuals, which could help identify subgroups who might benefit most from targeted noise-mitigation strategies. Enrichment in immune-related pathways, including interleukin signaling and chemotaxis, is consistent with prior work identifying vascular inflammation as a potential mechanism of noise-induced vascular damage. Regulation of different interleukin receptors suggests immunomodulatory effects of noise as already shown previously ^1, 2^ and changes in IL-24 expression, which is related to macrophage migration inhibitory factor mediated glucocorticoid regulation and TGF-β pathway, implies regulation of stress hormone signaling, tissue repair and immune cell proliferation and activation. Accordingly, IL-24 plays a role in HPA axis, cortisol reactivity and anxiety ^3^. TGF-β can signal through non-canonical pathways to activate extracellular signal-regulated kinase signaling, rat sarcoma homolog (Rho)-guanosine triphosphatase (GTPase) signaling, p38 mitogen-activated protein kinase (MAPK) signaling, c-Jun N-terminal kinase (JNK) signaling, nuclear factor-κB (NF-κB) signaling, phosphatidylinositol 3-kinase (PI3K)/AKR mouse thymoma proto-oncogene (AKT) signaling, as well as Janus kinase (JAK)/signal transducer and activator of transcription (STAT) signaling ^4^. S100A12 is linked to Toll-like receptor 7/8 cascade and innate immune system and chemokines CXCL6, CXCL11, CXCL1, CCL7 and CCL28 play a role in macrophage migration inhibitory factor mediated glucocorticoid regulation and TGF-β signaling as well as inflammatory pathways and chemotaxis. CD274, also called programmed cell death 1 ligand 1, represents an immune inhibitory receptor ligand that regulates T-cell activation. Nerve growth factor is important in the development and survival of neurons, especially those that transmit pain, temperature, and touch sensations (sensory neurons). Bone morphogenetic protein 6 is related to macrophage migration inhibitory factor mediated glucocorticoid regulation and TGF-β signaling. UDP-glucose glycoprotein glucosyltransferase 1 plays a role in quality control for protein transport out of the ER. Macrophage receptor with collagenous structure is a member of the scavenger receptors and part of the innate antimicrobial immune system, e.g. defending against Gram-positive and –negative bacteria. Sulfotransferase 1A1 (also called ST1A1) catalyze the sulfate conjugation of many hormones, neurotransmitters, drugs and xenobiotic compounds.

Besides the proteins that were identified as regulated in the network analysis, there are some more targets displaying at least a clear trend of change by noise. The chemokine CXCL1 plays a crucial role in immune responses, particularly in attracting neutrophils to sites of inflammation or injury. Glycosyltransferases may stimulate the active differentiation of M0 macrophages into M1 or M2 macrophages. Growth differentiation factor 2 (also called BMP-9) is highly related to TGF-β signaling and SMAD family transcription factors. Hydroxyacid oxidase 1 is involved in glyoxylate metabolism, glycine degradation and peroxisomal lipid metabolism. Heat shock protein 27 is related to stress response and acts as a protein chaperone and an antioxidant and plays a role in inhibiting apoptosis and actin cytoskeletal remodeling. Fc fragment of IgG receptor IIb participates in the phagocytosis of immune complexes and in the regulation of antibody production by B lymphocytes. Integrin subunit beta 1 binding protein 2 involves calcium ion binding and integrin binding. Poly (ADP-ribose) polymerase 1 apoptosis and survival via death receptor FAS signaling cascades and transcription-coupled nucleotide excision repair. Transferrin is an iron transport protein essential for keeping the free iron levels low and preventing oxidation reactions via free iron. Thrombopoietin regulates the production of platelets.

Comparative analysis of proteomic changes between participants with stronger endothelial responses to road traffic noise and those with minor changes of FMD in response to noise revealed a clear correlation of marker expression with the severity of endothelial function impairment (measured by FMD). Besides the targets mentioned above, IL-24, IL10R, CD274 (PD-L1), CX3CL1, MARCO, glycosyltransferase (GT) and CXCL1, some more were differentially regulated in the participants displaying high versus low FMD changes. Vascular endothelial growth factor D is active in angiogenesis and endothelial cell growth. CCL17 is a powerful chemokine produced in the thymus and by antigen-presenting cells like dendritic cells, macrophages and monocytes, attracting T-regulatory cells. CXCL5 stimulates the chemotaxis of neutrophils possessing angiogenic properties. Oncostatin M is a cytokine within the interleukin-6 protein family, produced by activated leukocytes. CCL23 is highly chemotactic for resting T cells and monocytes and slightly chemotactic for neutrophils. It also plays a role in macrophage migration inhibitory factor-mediated glucocorticoid regulation and TGF-β signaling. Neurotrophin-3 supports the survival of existing and growth of new neurons. The CD8A antigen is a cell surface glycoprotein found on most cytotoxic T lymphocytes that mediates efficient cell-cell interactions within the immune system. Renin is a part of the renin-angiotensin-aldosterone system involved in the regulation of blood pressure, and electrolyte balance.

These proteomic changes indicate that road traffic noise elicits heterogeneous responses in individuals, manifesting as immunomodulatory, stress-related, and vascular effects. Key alterations include regulation of interleukin receptors (e.g., IL-24, IL10R), chemokines (e.g., CXCL1, CCL17), and immune checkpoint molecules (e.g., CD274), pointing to involvement of stress hormone signaling, TGF-β pathways, and HPA axis function. Differential expression of proteins such as S100A12, MARCO, glycosyltransferases, and growth factors like VEGF-D and BMPs further supports changes in innate immunity, macrophage polarization, and endothelial function. Importantly, these proteomic signatures correlate with individual variability in endothelial response (FMD) to noise, linking immune and stress-related pathways to vascular health outcomes.

**Extended References**

1. Munzel T, Daiber A, Steven S, Tran LP, Ullmann E, Kossmann S, Schmidt FP, Oelze M, Xia N, Li H, Pinto A, Wild P, Pies K, Schmidt ER, Rapp S, Kroller-Schon S. Effects of noise on vascular function, oxidative stress, and inflammation: mechanistic insight from studies in mice. *Eur Heart J* 2017;**38**:2838-2849.

2. Eckrich J, Frenis K, Rodriguez-Blanco G, Ruan Y, Jiang S, Bayo Jimenez MT, Kuntic M, Oelze M, Hahad O, Li H, Gericke A, Steven S, Strieth S, von Kriegsheim A, Munzel T, Ernst BP, Daiber A. Aircraft noise exposure drives the activation of white blood cells and induces microvascular dysfunction in mice. *Redox Biol* 2021;**46**:102063.

3. Lipschutz R, Bick J, Nguyen V, Lee M, Leng L, Grigorenko E, Bucala R, Mayes LC, Crowley MJ. Macrophage migration inhibitory factor (MIF) gene is associated with adolescents' cortisol reactivity and anxiety. *Psychoneuroendocrinology* 2018;**95**:170-178.

4. Deng Z, Fan T, Xiao C, Tian H, Zheng Y, Li C, He J. TGF-beta signaling in health, disease, and therapeutics. *Signal Transduct Target Ther* 2024;**9**:61.
